# Diagnosis of Prostate Cancer through the Multi-Ligand Binding of Prostate-Derived Extracellular Vesicles and miRNA Analysis

**DOI:** 10.3390/life13040885

**Published:** 2023-03-27

**Authors:** Lidia Zabegina, Ilya Zyatchin, Margarita Kniazeva, Andrey Shalaev, Maria Berkut, Vladimir Sharoyko, Vladimir Mikhailovskii, Kirill Kondratov, Sergey Reva, Alexandr Nosov, Anastasia Malek

**Affiliations:** 1 Subcellular Technology Lab, Petrov National Medical Research Center of Oncology, 197758 Saint-Petersburg, Russia; 2 Department of Oncology No. 6, Pavlov First Medical State University, 197022 Saint-Petersburg, Russia; 3 Surgical Department of Oncourology, Petrov National Medical Research Center of Oncology, 197758 Saint-Petersburg, Russia; 4 Department of General and Bioorganic Chemistry, Pavlov First Medical State University, 197022 Saint-Petersburg, Russia; 5 Interdisciplinary Resource Center for Nanotechnology, Saint-Petersburg State University, 199034 Saint-Petersburg, Russia; 6 Translational Medicine Laboratory, City Hospital No. 40, 197706 Saint-Petersburg, Russia; 7 Oncosystem Ltd., 121205 Moscow, Russia

**Keywords:** prostate cancer, small extracellular vesicles (SEV), aptamer, miRNA, diagnosis

## Abstract

Background: The development of new non-invasive markers for prostate cancer (PC) diagnosis, prognosis, and management is an important issue that needs to be addressed to decrease PC mortality. Small extracellular vesicles (SEVs) secreted by prostate gland or prostate cancer cells into the plasma are considered next-generation diagnostic tools because their chemical composition might reflect the PC development. The population of plasma vesicles is extremely heterogeneous. The study aimed to explore a new approach for prostate-derived SEV isolation followed by vesicular miRNA analysis. Methods: We used superparamagnetic particles functionalized by five types of DNA-aptamers binding the surface markers of prostate cells. Specificity of binding was assayed by AuNP-aptasensor. Prostate-derived SEVs were isolated from the plasma of 36 PC patients and 18 healthy donors and used for the assessment of twelve PC-associated miRNAs. The amplification ratio (amp-ratio) value was obtained for all pairs of miRNAs, and the diagnostic significance of these parameters was evaluated. Results: The multi-ligand binding approach doubled the efficiency of prostate-derived SEVs’ isolation and made it possible to purify a sufficient amount of vesicular RNA. The neighbor clusterization, using three pairs of microRNAs (miR-205/miR-375, miR-26b/miR375, and miR-20a/miR-375), allowed us to distinguish PC patients and donors with sensitivity—94%, specificity—76%, and accuracy—87%. Moreover, the amp-ratios of other miRNAs pairs reflected such parameters as plasma PSA level, prostate volume, and Gleason score of PC. Conclusions: Multi-ligand isolation of prostate-derived vesicles followed by vesicular miRNA analysis is a promising method for PC diagnosis and monitoring.

## 1. Introduction

Prostate cancer (PC) is one of the most common malignant diseases and the second leading cause of cancer death in men. This justifies the ongoing effort of the scientific and biotechnological community to improve existing approaches to screening, diagnosis, and management of PC. Screening for PC is still based on evaluation of plasma PSA levels; however, its rationale is still controversial [1]. The bases for PC diagnosis are PSA assessment, digital rectal examination (DRE), and biopsy [2]. The management of PC is based on the stratification of the patient into different risk groups taking into account the PSA level, Gleason score, and the spread of the tumor [3,4]. There have been a number of new serum-, urine-, and tissue-based PC biomarkers developed over the last decade [5,6]. The most advanced results have been obtained by deep sequencing of cell-free DNA (cfDNA) using custom panels that allow analysis of PC-related genes [7,8]. However, these findings still have limited clinical applications. Small extracellular vesicles (SEVs) of endosomal origin, or exosomes, are considered a “next-generation diagnostic tool” in PC [9,10,11,12]. A body of experimental evidence indicated that SEVs secreted by the prostate epithelial cell or PC cells may contain specific molecular markers such as membrane proteins [13,14], messenger RNA [15], or microRNA [16]. Evaluation of these exosomal components opened up possibilities to develop new non-invasive techniques for PC diagnosis and management. The first solution for the analysis of ERG, PCA3, and SPDEF transcripts within urine-derived SEVs (ExoDx Prostate—IntelliScore) was released in 2016 [17]. Impact of this test on the management of patients with elevated PCA (2-10 ng/mL) was evaluated in clinical trials (NCT02702856; NCT03031418).

In contrast to urinary exosome assessment, the analysis of plasma SEVs is rather challenging because plasma is a protein-enriched liquid containing a huge number of vesicles derived from various tissues. The important advantage of SEVs is that their surface may contain tissue-specific markers, and the specific population of vesicles can be isolated from plasma using ligands to such markers. Proof-of-concept studies were performed in 2008 when tumor-derived exosomes were enriched by a modified magnetic activated cell sorting procedure, using anti-epithelial cell adhesion molecule (EpCAM) [17]. In our previous studies, we demonstrated the possibility to isolate TPO(+)SEV using super-paramagnetic beads (SPMB) functionalized with antibodies against thyroperoxidase (TPO) [18]. It was shown that TPO(+)SEVs derived from the thyroid gland and miRNA lt-7 from this population of SEVs are potential markers of follicular thyroid cancer. Next, we improved the tissue-specific SEV isolation technique using DNA-aptamers instead of antibodies, and analysis of miRNA in PSMA(+)SEV was evaluated as a new approach for PC diagnosis [19].

However, considering the size of the prostate, the prostate-derived SEVs can make up an extremely minor fraction of the total highly heterogeneous mixture of plasma vesicles. We assume that the efficiency of prostate-derived SEV isolation is the “bottleneck” of the proposed technology. In the present study, we attempted to address this issue using the composition of DNA-aptamers binding with various prostate-specific determinants of the SEVs’ surface. The most effective DNA-aptamers were selected using an AuNP-aptasensor [20], and aptamers were fixed on the surface of SPMB through the click chemistry; PC-associated miRNA from the isolated SEVs were analyzed with two-tailed RT-PCR. The developed analytic approach allowed us to distinguish PC patients and healthy donors. Moreover, obtained results were associated with plasma PSA level, prostate volume, and Gleason score that further indicated the clinical relevance of the miRNA pattern of isolated SEVs.

## 2. Materials and Methods

### 2.1. Patients

Blood samples were obtained from patients undergoing treatment at the Department of Oncourology at the Petrov NMRC of Oncology (n.13) and the Clinic of Urology at the Pavlov First Saint-Petersburg State Medical University (n.36). Control samples of blood were collected from healthy donors (n.18) of corresponding age at the Blood Transfusion Department of N.N. Petrov NMRC of Oncology. The study was planned according to the guidelines of the Declaration of Helsinki and was approved by the Ethics Committee of the Petrov NMRC of Oncology N°1 from 04.02.2021. Patients and donors gave informed consent to participate in the study. Plasma samples from 13 patients with advanced (metastatic) PC were used to test the technology. These patients were on palliative care at the N.N. Petrov NMRC of Oncology. The study included also patients with a localized histologically confirmed PC who met the specified criteria: age 50–74 years (median age 66.5); no chronic or metabolic diseases; PSA level > 4 ng/mL (median PSA 9.75 ng/mL); prostate volume 4–80 cm³ (median prostate volume 41.05 cm^3^); PC stage: T2–T3N0M0; and Gleason score: 6–9. According to standard criteria, PC patients were distributed into three risk groups [4]. Plasma samples from these patients were used to estimate the diagnostic value of the developed method. Detailed information about patients with localized PC are presented in Supplementary Table S1A. Information regarding the biological material used in the study is summarized in Supplementary Table S1B.

### 2.2. Plasma Sampling and SEV Isolation

Venous blood samples (5 mL) were collected into BD Vacutainer EDTA Tubes, and plasma was immediately isolated using centrifugation for 15 min at 1500× *g*, then aliquoted, frozen, and stored at −80 °C. Before use, the frozen plasma was slowly thawed at 4 °C, sequentially centrifuged at 300× *g* for 10 min, 1500× *g* for 10 min, and 10,000× *g* for 10 min, and filtered through a 0.2 µm syringe filter to remove cellular debris. Prepared plasma is hereafter referred to as pellet pure plasma (PPP) and was used for all experiments. The total SEV population was isolated from PPP using a two-phase polymer system (TPPS) as previously described [21]. Briefly, a solution of dextran (450–650 kDa, 1.5%) and polyethylene glycol (35 kDa, 3.5%) (both Sigma-Aldrich, St. Louis, MO, USA) was prepared in the 2 mL of PPP, and the same amounts of polymers were dissolved in an equal volume of phosphate-buffered saline (PBS) to prepare a protein-depleting solution (PDS). For phase separation, the solution was centrifuged for 10 min at 1000× *g*, after which the upper phase was replaced with PDS, and the solutions were well mixed and centrifuged again. The upper phase was removed, and the lower phase containing SEVs was resuspended in 100 μL of PBS.

### 2.3. Maintaining of PC Cell Cultures and SEV Isolation

For this study, we used DU145 and PC-3 prostate cancer cell lines. Cells were maintained in RPMI1640 medium, supplemented with 10% FBS and antibiotics. Cells were incubated at 37 °C in a CO_2_ incubator at 5% CO_2_. The total SEV population was isolated from DU145 and PC-3 cell culture media by double ultracentrifugation at 110,000× *g* for 8 h and 2 h, respectively, as described [22].

### 2.4. SEV Labelling with CM-Dil

An amount equal to 2 μL of a 50 μM Vybrant™ DiI Cell-Labeling Solution (Thermo Fisher Scientific, Waltham, MA, USA) diluted in DMSO (Biolot, Saint-Petersburg, Russia) was added to 2 mL of the PPP followed by incubation at 37 °C for 20 min with moderate stirring. PURE-EV size exclusion chromatography columns (HansaBioMed, Tallinn, Estonia) were used for SEV purification/isolation according to the producer’s protocol, with slight modifications [23]. Briefly, 2 mL of the sample was loaded into the SEC column, and 23 fractions of 500 µL were collected. Fractions 9–11 were used for further experiments.

### 2.5. Nanoparticle Tracking Analysis (NTA)

The size and concentration of the isolated vesicles were measured using a NanoSight NS300 analyzer (Malvern Panalytical, Malvern, UK) at camera level: 9, shutter slider: 440, slider gain: 24, and threshold level: 5. Each sample was pumped through the observation camera to record five measurements for 30 s for 749 frames at different microvolumes of the same sample. Based on the results of the five measurements, the values of the size and concentration of the nanoparticles were averaged.

### 2.6. Low-Voltage Scanning Electron Microscopy (LVSEM)

Visualization was performed using low-voltage scanning electron microscopy (LVSEM) on a scanning electron microscope with high-resolution Merlin (Carl Zeiss, Jena, Germany) at the Interdisciplinary Resource Center for Nanotechnology of St. Petersburg State University with the standard technique described previously [24].

### 2.7. Bead-Assisted Flow Cytometry of the Total SEV Population

To confirm the exosomal nature of TPPS-isolated SEVs, the membrane tetraspanins were assayed by flow cytometry using an Exo-FACS kit (HansaBioMed, Tallinn, Estonia) according to the manufacturer’s protocol. SEVs were absorbed nonspecifically to the surface of latex microparticles and stained with antibodies conjugated with fluorescent labels PE (CD9, BioLegends, San Diego, CA, USA), FITC (CD63, Abcam, Cambridge, UK), and APC (CD81, BioLegends, San Diego, CA, USA). All measurements were performed on a Cytoflex Platform (Beckman Coulter, Brea, CA, USA).

### 2.8. Aurum Nanoparticle and AuNP-Aptasensor

The structure and the operation principle of the Aurum Nanoparticle (AuNP)-aptasensor were described in detail [20]. Nanoparticles were synthesized as previously described [25]. Sequences of used DNA-aptamers with an affinity to prostate cancer cells were evaluated previously (Supplementary Table S2), whereas the synthesis was performed by Lumiprobe Ltd. and Syntol Ltd. (both Moscow, RF). To form AuNP-DNA complexes, the AuNP suspension (10 μL at concentration of 2 × 10^15^ particles/mL) was mixed with 2.5 μL of aptamer solution (at concentration 100 pmol/μL) and incubated for 30 min at 4 °C. Next, 1 μL of the total SEV population (concentration 10^8^ /mL) was added to 12.5 μL AuNP–DNA complexes. The restored peroxidase activity of AuNPs was evaluated after adding 10 μL of 3,3′,5,5′-tetramethylbenzidine, TMB (Xema Co. Ltd., Moscow, Russia) to the resulting solution and incubated for 18 min at 37 °C in the dark. Finally, the suspension was centrifuged (6000× *g*, 3 min), the supernatants were carefully transferred to the wells of a 384-well plate, and the absorption spectra were measured immediately using a Varioskan LUX Multimode Microplate Reader (Thermo Fischer Scientific, Waltham, MA, USA). Distilled water was used as a blank, and the peroxidase activity of unmodified AuNP was evaluated as positive control (maximum of peroxidase activity), whereas AuNP-DNA complex (without SEVs) was used to set up a minimum of peroxidase activity of completely inhibited AuNP. Additionally, two aptamers, binding CD63 and CD30 membrane proteins, were used as controls. The absorbance spectra were investigated in diapason from 200 to 500 nm.

### 2.9. Formation of SPMB-Apt Complexes for Prostate-Derived SEV Isolation

The complex was composed of super-paramagnetic beads (SPMBs) with the surface functionalized by -N3 groups (Click Chemistry Tools, Phoenix, AZ, USA) and DNA-aptamers (Apt) modified by a dibenzocyclooctyne group (DBCO) at the -5′ end. Complex SPMB–Apt was formed by the reaction of azide-alkyne cycloaddition as described [19]. First, 1 µL of SPMBs (1 mg/mL) was incubated in a 200 µL I-Block™ Protein-Based Blocking Reagent (ThermoFisher Scientific, Walthman, MA, USA) at 4 °C for 1 h to prevent nonspecific binding. The SPMBs were then washed and re-suspended in 100 µL of PBS. Then, 1 µL of Apt solution (100 pM) was added to the suspension of blocked SPMB, mixed, and incubated for 3 h at RT. The SPMB-Apt complexes were washed twice with 200 µM of PBS to remove the unbound aptamers.

To achieve binding of specific population of SEVs, 100 µL of the total population of plasma SEVs (concentration 2 × 10^11^ particles/mL) were added to the pellet of SPMB-Apt complexes, mixed, incubated at 4 °C overnight under moderate stirring, and washed twice in 200 µL of unbound SEVs. In order to test the efficacy of specific SEV binding, the total population of plasma vesicles was labelled in advance with lipophilic dye CM-Dil, purified by size exclusion chromatography columns (Section 2.4) incubated with SPMB-Apt, then washed and assayed by flow cytometry. In order to purify vesicular RNA, TPPS-isolated SEVs were incubated with SPMB-Apt, and the SPMB-Apt-SEVs complexes were washed, pelleted, and mixed with lysis buffer.

### 2.10. RNA Isolation and RT-PCR

RNA from the SPMB-bound SEVs (SPMB-Apt-SEVs) was isolated with an RNAGEM kit (MicroGem, Dunedin, New Zealand) according to the manufacturer’s protocol. The isolated RNA was analyzed by two-tailed reverse transcription (RT) followed by real-time polymerase chain reaction (PCR) using corresponding kits ALMIR series (Algimed Techno, Minsk, Belarus) according to the manufacturer’s protocol. An amount equal to 2 μL of the total RNA solution was used for the RT reaction in a volume of 20 µL with 0.05 pM of two-tailed RT-primer and 100 U of M-MuLV–RH reverse transcriptase and corresponding buffer. Reaction conditions: 25 °C—45 min, 85 °C—5 min. Then, 4 μL of RT reaction mix were used for PCR in 20 µL with 0.06 pM of each PCR primer, 0.04 pM of FAM-labelled probe, and 10 µL of 2xPCR master mix composed of Taq DNA Polymerase, dNTPs, and corresponding buffer. Reaction conditions: 5′—95 °C and 40 cycles of 5″—95 °C/15″—60 °C. All reactions were conducted in technical triplicates, and results were obtained with CFX Manager Software 3.1. Instead of the standard approach for PCR data normalization using reference molecules, amplification ratios of all miRNA pairs were estimated as 2^Ct(miR−A) − Ct(miR−B)^ and used as diagnostic criteria. 

The experimental data were processed using Nanosight NTA 3.4, CytExpert software, OriginPro 9.1, and Graph Pad Prizm 8. Statistical differences between groups of samples (healthy donods (n.8) vs. PC patients (n.13)) were evaluated using the nonparametric Mann–Whitney test. ROC analysis was used to assess the diagnostic significance of the developed method in the group of healthy donors (n.18) vs. various groups of patients with different clinical characteristics (the size of the groups varied from n.3 to n.36).

## 3. Results

### 3.1. Characteristics of Small Extracellular Vesicles (SEVs) from Plasma

**Figure 1:**
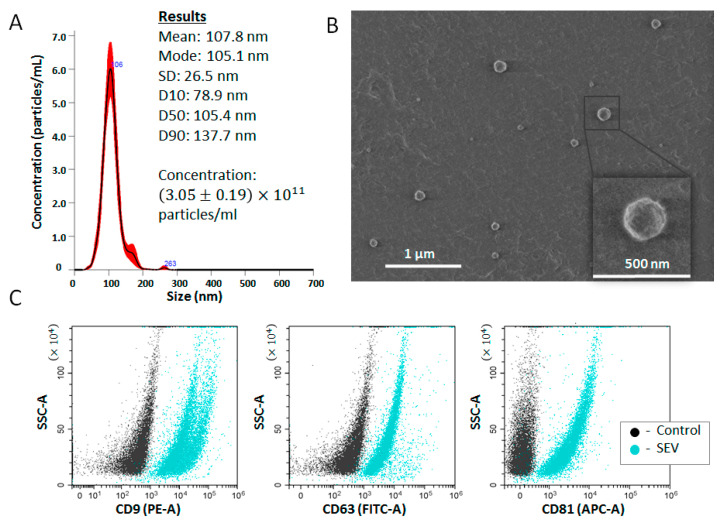
Characteristics of EVs isolated using the two-phase polymer system (TPPS). (**A**) Representative examples of NTA of SEVs isolated from donor’s plasma. (**B**) SEVs visualized using low-voltage scanning electron microscopy. (**C**) Exosomal markers assayed on the SEV surface using on-bead flow cytometry. SEVs nonspecifically attached to latex beads were stained with fluorescent-labelled antibodies.

The total SEV population was isolated from all plasma samples with TPPS [20]. The size of the SEVs measured by the NTA was in a range from 75 to 140 nm with a mean size of 110 nm. The concentration of isolated SEVs varied from 1.83 to 3.04 × 10^11^ particles/mL. A representative example of NTA for one sample is shown in Figure 1A. The morphology of the isolated SEVs was assayed using LVSEM; Figure 1B demonstrates spherical particles of various sizes. It was expected that results of NTA and LVSEM varied slightly due to the difference in sample preparation and measurement principles. Exosomal markers, tetraspanins CD9, CD63, and CD81, were detected on the surface of isolated vesicles using on-bead flow cytometry (Figure 1C). We did not observe a difference between SEVs isolated from the plasma of PC patients and those from healthy donors for any of the parameters tested.

The obtained results indicated that the population of particles isolated from plasma with TPPS was composed of small extracellular vesicles (SEVs), and at least some of these vesicles were positive for exosomal markers. These results corresponded well with previously published data [21]. We did not attempt to isolate a pure fraction of exosomes, and our further experiments were performed with the obtained population of SEVs.

SEVs secreted by PC cells (DU145 or PC-3) were isolated from 300 mL conditioned media and quantified by NTA. The concentration of SEVs was then adjusted to 2 × 10^11^ particles/mL.

### 3.2. Selection of Most Effective DNA-Aptamers

On the basis of scientific data analysis, we chose eight DNA aptamers to selectively bind to known or unknown markers associated with prostate or PC cells (Supplementary Table S2). The affinity of these aptamers for prostate or PC cells was evaluated by AuNP-aptasensor, as developed previously [20].

**Figure 2:**
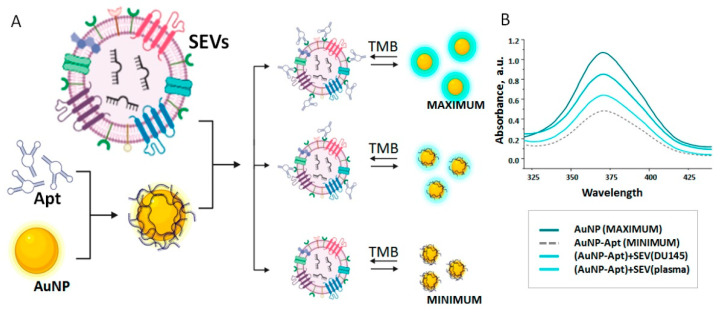
Principle of AuNP-aptasensor operation. (**A**) Schematic presentation of components’ interaction. (**B**) Readout of system from maximum enzymatic activity (free AuNP) to minimum enzymatic activity (AuNP inhibited by attached Apt4) after peroxidation of TMB. Presence of SEVs resulted in replacement of aptamers from the surface of NPs to the surface of vesicles. The partial restoration of NP enzymatic activity was evaluated after peroxidation of TMB and absorbance measurement.

First, we thought to estimate the operating range of the AuNP-aptasensor. The principle of the analytical procedure is schematically presented in Figure 2A: free AuNPs have peroxidase activity, which can be assessed by the TMB peroxidation color reaction. Attachment of DNA-aptamers to the surface of AuNPs reversibly inhibited their peroxidase activity. We measured the activity of free AuNPs (AuNP (MAXIMUM)), and the activity of AuNPs completely inhibited any of the DNA-aptamers (AuNP-Apt (MINIMUM). The difference between these parameters provided us with a working diapason of AuNP-aptasensors that varied slightly between the DNA-aptamers due to difference in their length and ability to shield the surface of Au nanoparticles.

It is expected that the appearance of SEVs bearing prostate-associated surface markers in the reaction mixture will lead to the detachment of aptamers from the surface of AuNPs and the restoration of their peroxidase activity within a working range (Figure 2B). The intensity of such a restoration depends on the affinity of the interaction between the SEV surface markers and the DNA-aptamers, which is determined by the sequence and spatial conformation of aptamers. To evaluate the properties of selected aptamers, we used AuNP-aptasensors equipped with each of them [20] and SEVs secreted by either prostate cancer cells (DU145, PC3) or by SEVs isolated from plasma of healthy donors (n.8). In order to minimize inter-individual variability of plasma SEV characteristics, we used samples of SEVs from eight healthy donors randomly selected from our collection, normalized by concentration and pooled. It was expected that PC cell-derived SEVs will more actively attract the aptamers and liberate the surface of AuNPs than SEVs isolated from donor’s plasma.

Representative results of plasma SEVs and SEVs secreted by DU145 cells assayed by AuNP-aptasensor with Apt4 are shown in Figure 2B. Eight aptamers were used to assay tree types of SEVs (plasma, DU-145, and RC-3-derived), and the degree of restored peroxidase activity of AuNP was expressed as a percentage of the operating range (Table 1).

The DNA-aptamers that reacted less with plasma SEVs (lower % of the restored peroxidase activity) and more actively reacted with SEVs secreted by cultured PC call DU-145 (higher % of the restored peroxidase activity) were supposed to be suitable for further experiments. Five DNA-aptamers (Apt4, Apt5, Apt6, Apt7, and Apt8) revealed a large difference between the signal obtained with PC cell-derived SEVs and the signal obtained with plasma SEVs (Delta > 50% of AuNP-sensor working range). These aptamers were selected for further work.

### 3.3. Relative Quantification of PC-Derived SEVs in Plasma of Healthy Donors and PC Patients

**Figure 3:**
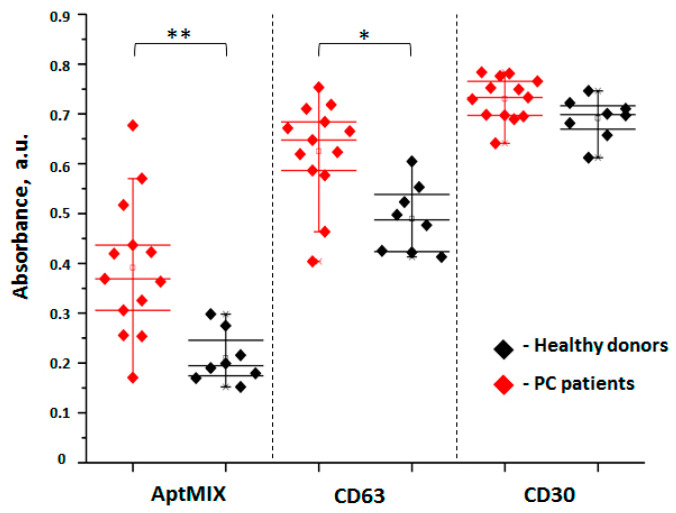
Assessment of prostate-derived SEVs in plasma of patients with advanced PC and healthy donors using an AuNPaptasensor equipped with a prostate-specific aptamer mixture (AptMIX), CD63-specific aptamer, and CD30-specific aptamers. The absorbance-reflecting number of prostate- or PC-derived SEVs was measured at 350 nm. The statistical significance of the difference between the groups was estimated by Mann-Whitney test: * (*p* < 0.05), ** (*p* < 0.005).

Next, we attempted to see whether development of PC is associated with an increase in the concentration of SEVs derived from prostate and/or PC cells. The total populations of plasma SEVs were isolated from the plasma of healthy donors and patients with advanced (metastatic) PC. In order to assay the prostate- or PC-derived plasma SEVs with maximal efficacy, the AuNP-aptasensor was equipped with five selected DNA-aptamers mixed in equimolar ratio (AptMIX), while the total amount of DNA was kept as usual (2.5 μL of solution at concentration 100 pmol/μL). Measurements were carried out the same way as in previous experiments. Results obtained from the samples of healthy donors’ plasma (n = 8) and those of PC patients (n = 13) are presented in Figure 3. In parallel, we assayed a quantity of CD63(+)SEVs and CD30(+)SEVs as control using corresponding DNA aptamers described previously [20].

A statistically significant difference between the groups of PC patients and healthy donors was obtained using aptMIX (*p* < 0.005). An amount of CD63(+)SEVs was also slightly increased in the plasma of PC patients compared to healthy donors (*p* < 0.05). Since exosomal marker CD63 is expressed in various types of solid tumors including PC [26], this may explain the increase in CD63(+)SEVs in patients with PC. We did not observe a difference in the concentration of CD30(+)SEVs between analyzed groups. The results confirmed that the appearance of a specific prostate-derived (Apt4(+) and/or Apt5(+) and/or Apt6(+) and/or Apt7(+) and/or Apt8(+)) population of SEVs is associated with the development of PC. Even though we have no data to estimate how large and homogeneous this population is, we can suppose that the miRNA content of these vesicles may better reflect PC development than the total population of plasma SEVs. Assuming a prostatic origin if an isolated SEV population, we further referred to them as pSEV.

### 3.4. Isolation of pSEVs with Functionalized SPMP

**Figure 4:**
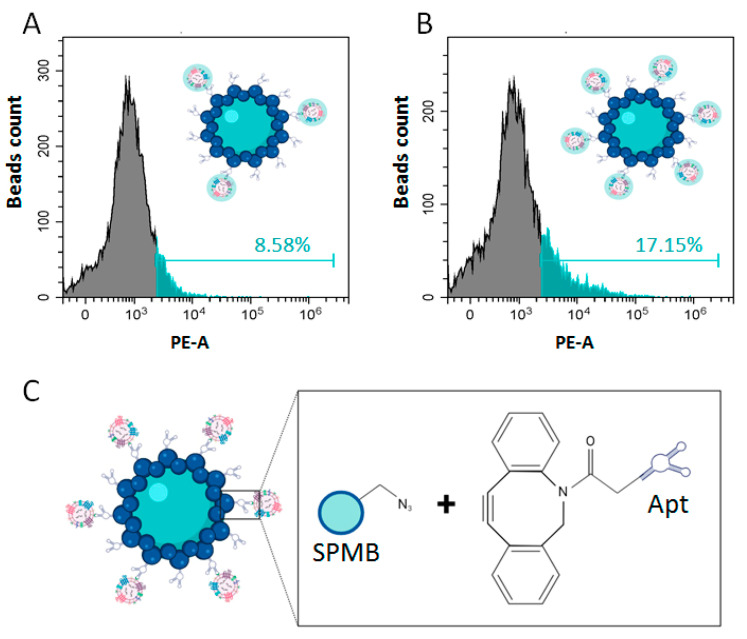
Efficacy of prostate-specific SEVs’ sorption. (**A**) The scheme of super-paramagnetic beads’ (SPMB) functionalization with aptamers through click chemistry. To evaluate the efficacy of prostate-derived SEVs’ binding, vesicles were labelled lipophilic dye CM-Dil, incubated with SPMBs functionalized with single aptamer Apt4 (**B**) or with a mix of five different aptamers—AptMIX (**C**). SPMB with attached CM-Dil-labelled SEVs were assayed with flow cytometry.

The next experiment was aimed at comparing the efficiency of the isolation of pSEVs with SPMB functionalized using a single anti-PSMA-aptamer Apt3 [27] used previously [19] or using a mix of five aptamers—AptMIX. The mechanism of SPMB functionalization via click chemistry is schematically presented in Figure 4A.

SPMB were prepared with a standard protocol [19]. Plasma SEVs from healthy donors (n. 8) were labelled with the membrane dye Vibrant Dil (CM-Dil), purified with SEC [23], pooled, and incubated with SPMB functionalized with either Apt3 or AptMIX. Relative quantification of isolated pSEVs was performed with flow cytometry; results are presented in Figure 4B,C. Use of SMPB functionalized using a combination of Apts (AptMIX) almost doubled the isolation efficiency of SEVs (8.58% vs. 17.15%). These results confirmed the efficiency of the multi-ligand binding approach for the isolation of pSEVs.

### 3.5. Evaluation of Diagnostic Value of PC-Associated miRNA in pSEVs

**Figure 5:**
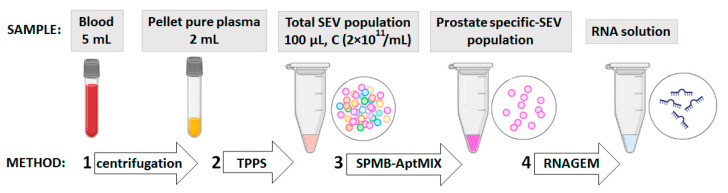
Schematic workflow of pre-analytic procedure. Successive stages are indicated by numbers.

The pSEVs were isolated from the plasma of PC patients (n = 36) and healthy donors (n = 18) using SPMB-AptMIX complex according to the previously described protocol. Patients included in this study had histologically confirmed local PC with neither lymph node nor distant metastases detected (Supplementary Table S1A). The schematic workflow of the applied pre-analytic procedure is presented in Figure 5.

In order to investigate the association between pSEV miRNA content and the clinical characteristics of PC, patients with localized PC were grouped according to a three-tiered sub-classification system [28] (Supplementary Table S1). The microRNA associated with PC were selected on the basis of our previous studies and PubMed search (Supplementary Table S3). After total RNA extraction, a semi-quantitative assessment of the selected miRNAs concentrations was conducted using RT-PCR. A complete list of averaged Ct is presented in Supplementary Table S4.

**Figure 6:**
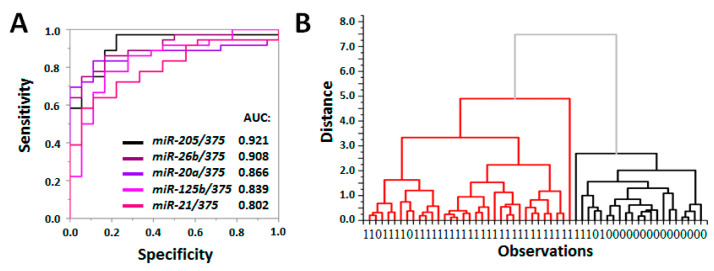
Distinguishing of PC patients and healthy donors by the assessment of miRNAs in prostate-derived SEVs. (**A**) ROC analysis for five reciprocally dysregulated miRNA pairs with AUC > 0.8. (**B**) Dendrogram obtained by furthest neighbor clustering method using values of amplification ratio for three pairs of microRNAs: miR-205/miR-375, miR-26b/miR375, and miR-20a/miR-375. Two main clusters are shown by red and black.

In our study, we assayed 12 PC-associated miRNA molecules in specific pc-SEVs isolated from 54 blood samples (PC n.36 and healthy donors n.18). It was doubtful to be able to select the appropriate reference miRNA and to perform data normalization. The evaluation of the amplification ratio (amp-ratio) of two simultaneously and reciprocally dysregulated miRNAs is a promising approach for miRNA expression data interpretation validated in several studies by us [28] and others [29]. Here, the amp-ratio was obtained using the following formula: Ratio = 2 ^Ct(miR-A) − Ct(miR-B)^ for each possible pair of miRNAs (Supplementary Table S5). The amp-ratios were explored as diagnostic parameters to distinguish samples of PC patients and healthy donors, and the efficiency of such diagnosis was assessed using ROC analysis. Figure 6A shows the ROC curves for the five microRNA pairs with the highest AUC values; the results of other miRNA pairs evaluation are presented in Supplementary Table S6.

The statistically significant difference between PC and control groups and the AUC > 0.8 indicated a high diagnostic value of certain miRNA pairs assayed in the population of pSEVs. To confirm this, we used cluster analysis. We took the ratio for three pairs of microRNAs (AUC > 0.85) (miR-205/miR-375, miR-26b/miR375, miR-20a/miR-375) and applied the furthest neighbor clustering method. The resulting dendrogram is shown in Figure 6B: samples obtained from PC patients and healthy donors were distinguished with sensitivity—93.94%, specificity—76.19%, and accuracy—87.04%. Obtained results confirmed the high diagnostic potency of miRNAs’ analysis in a specific pSEV population.

### 3.6. Clinical Significance of PC-Associated miRNA in pSEVs

**Figure 7:**
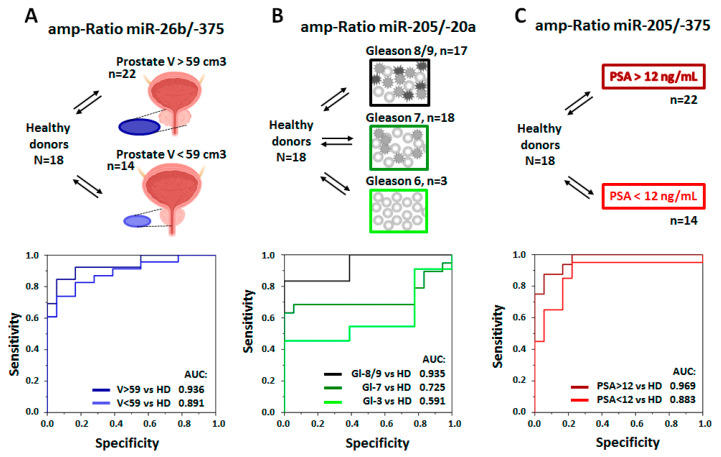
Example of the distinguishing of healthy donors from PC patients with different extents of clinical characteristics using amp-ratio values. The number of patients in each group is indicated. (**A**) Healthy donors were distinguished from PC patients with different prostate sizes measured using MRI (V > 59 cm^3^, n.22; V < 59 cm^3^, n.14). Diagnostic criterion: miR-26b/-375 amp-ratio. (**B**) Healthy donors were distinguished from PC patients with different Gleason scores of prostate cancer evaluated histologically (Gleason score 6, n.3; Gleason score 7, n.18; Gleason score 8/9, n.17). Diagnostic criterion: miR-205/-20a amp-Ratio. (**C**) Healthy donors were distinguished from PC patients with different levels of plasma PSA (PSA > 12 ng/mL, n.22; PSA < 12 ng/mL, n.14). Diagnostic criterion: miR-205/-375 amp-Ratio.

The personalized management of PC patients should be based on an integral evaluation of different disease markers. For instance, assessment of the plasma PSA level, the size/spread of the tumor, and the Gleason score range makes it possible to stratify patients into groups with various risks of disease progression [4,28]. If miRNAs are considered hub-regulators of altered protein synthesis in PC cells, the analysis of miRNA in pSEVs should reflect disease status. We attempted to prove this hypothesis by testing the correlation between the value of reciprocally regulated miRNAs’ amp-ratio and standard clinical parameters (the plasma PSA level, the volume of prostate, and the Gleason score). To do this, all possible miRNA pairs’ amp-ratios were explored as the criteria for the differentiation of healthy donors from groups of patients with different ranges of risk factors. Diagnostic values were estimated using ROC analysis, and a complete list of the results (AUC values) is presented in Supplementary Table S7. The most interesting results are demonstrated in Figure 7.

For instance, when the amp-ratio of miR-26b/-375 was used as a criterion to differentiate healthy donors from PC patients (Figure 7A), the efficiency of such differentiation was higher for the group of patients with a prostate size over 59 cm^3^ (AUC = 0.936) than for the group of patients with a prostate size up to 599 cm^3^ (AUC = 0.891). A similar tendency was observed for the amp-ratio of miR-205/miR-20a (Figure 7B): the healthy donors were differentiated from patients with high-grade PC (Gleason score 8/9) with AUC = 0.935, from patients with medium-grade PC (Gleason score 7) with AUC = 0.739, whereas the difference between healthy donors and patients with low-grade PC (Gleason score 6) was negligible (AUC = 0.625). The amp-ratio of miR-205/miR-375 (Figure 6C) can be used to differentiate healthy donors from PC patients more precisely when plasma PSA > 12 ng/mL (AUC = 0.969) and less precisely when plasma PSA < 12 ng/mL (AUC = 0.883). The results of other miRNA pairs’ evaluation are presented in Supplementary Table S7. Altogether, obtained results indicate a high predictive value of miRNA isolated from pSEV.

## 4. Discussion

MiRNAs have been explicitly investigated for their potential to serve as molecular markers for PC [30]. Several recent studies have demonstrated the great potential of plasma circulating miRNAs: miR-4289, -326, -152-3p and -98-5p [31], miR-21, -125b, 126, -141, -375, -let-7b [32], miR-150-5p [32], miR-4732-3p, -98-5p, -let-7a-5p, -26b-5p, -21-5p [33] and others; however, results rarely overlap. The potential of using circulating miRNA to diagnose PC has recently been reviewed with cautious optimism [34]: “there are a lot of pitfalls in our knowledge”. The most important factors hampering development of miRNA-based liquid biopsy for PC are (1) the complex and poorly characterized composition of plasma mRNAs; (2) the little effect that prostate-derived miRNAs can have on the plasma miRNA profile; and (3) the imperfection of miRNA isolation and quantification techniques. The plasma SEVs are considered natural vehicles of miRNA, which might provide an excellent option to solving most of the concerns mentioned and developing a miRNA-based PC diagnostic tool.

However, simple isolation of plasma SEV followed by analysis of vesicular miRNA is not enough. As it was demonstrated by quantitative and stoichiometric analysis of the miRNA content of plasma exosomes [35], most of the vesicles derived from standard preparation do not harbor many copies of miRNA molecules. Muneesh Tewari et al. [35] quantified SEV and PC-relevant miRNA-126 and -223 in the plasma of healthy donors and PC patients. Thus, the number of SEV varied in a rather narrow diapason 1–4 × 10^9^/µL, and the number of miR-126 varied in wide diapason within 10^4^–10^5^/µL, while the number of miR-223 varied within 10^5^–10^6^/µL. This means that marker miRNAs were found in one out of 10^5^ or 10^3^ analyzed vesicles only. In this case, the total concentration of circulating vesicles and the efficiency of their isolation will crucially influence the results of miRNA quantification. To the best of our knowledge, these results have not been directly confirmed by anyone, but they explain quite well the poor agreement between exosomal microRNA studies published so far [31,32,33,34].

Considering the variability of the plasma SEV population and extremely rare distribution of the specific miRNAs within SEVs, the isolation of tissue- or cell-specific SEVs seems to be the obligatory step prior to vesicular miRNA assessment. Foroni et al. explored this assumption using immunoadsorption of the specific SEV population using patented antibodies against undisclosed surface marker of cells growing in hypoxic conditions [15]. If the cells of PC suffer from hypoxia, their vesicles can bear this marker and can be enriched via immunoadsorption. Usssing the SoRTEV RNA enrichment protocol, the authors demonstrated an increased sensitivity of detection of the disease-associated splicing variant of the androgen receptor (AR) associated with the response to antiandrogen therapy. Another hypothesis was explored in our study. We supposed that malignant transformation does not always and straightaway lead to complete dedifferentiation of cancer cells, and most of the tumors keep tissue-specific surface markers. This has been demonstrated in colon cancer [36]. Over the last year, we developed and optimized the technology of tissue-specific SEV isolation based on superparamagnetic beads’ functionalized by ligand to tissue-specific markers [18,19]. In the present study, we explored a new strategy of multi-ligand binding of tissue-specific SEVs using DNA-aptamers developed by other research groups (References in Supplementary Table S2). Results obtained in our study justified this approach.

However, it should be taken into account that multi-ligand binding should increase efficiency but could reduce specificity of tissue-specific SEV isolation. Thus, the development of aptamers with an affinity to tissue-specific cell surface markers will be a next step in our research. Moreover, deep profiling of miRNA in such tissue-specific population of SEVs needs to be performed for identification of the most relevant diagnostic markers. Finally, an evaluation of the diagnostic performance of the developed method should be performed through the analysis of large cohorts of PC patients, patients with benign prostatic hyperplasia (BPH), and healthy donors.

## 5. Conclusions

MiRNA in the prostate-specific plasma SEV population (pSEV) has great diagnostic potential. A pSEV can be effectively isolated using super-paramagnetic beads functionalized with multiple DNA-aptamers. The profile of miRNA in pSEVs is of great diagnostic value and is associated with PSA level, volume of prostate, and Gleason score of PC.

## Figures and Tables

**Table 1:** Comparative evaluation of DNA-aptamers’ (Apt) affinity to SEVs from either plasma or PC cell cultures (DU-145, PC3) assayed by AuNP-aptasensor and expressed as a percentage of the total operating range.

	Apt1	Apt2	Apt3	Apt4	Apt5	Apt6	Apt7	Apt8
SEV (plasma)	6.61	19.91	17.53	**7.15**	**6.34**	**10.69**	**14.78**	**8.96**
SEV (DU-145)	36.9	77.7	63.87	**63.24**	**81.42**	**72.63**	**73.83**	**63.49**
SEV (PC-3)	62.42	60.76	55.37	**60.46**	**64.89**	**63.7**	**63.83**	**67.49**
Delta ^1^	43.05	49.32	42.09	**54.7**	**66.82**	**57.48**	**54.05**	**56.53**

^1^ Delta = SEV (plasma)—averaged (SEV (du-145), SEV (PC-3)).

## Data Availability

All data are presented in the Supplementary Materials. Authors will be happy to provide any additional information or technical details.

## Supplementary Materials

The following supporting information can be downloaded at: https://www.mdpi.com/article/10.3390/life13040885/s1. Table S1A: Information about patients. Table S1B Biological material used for SEV isolation and analysis. Table S2: DNA-aptamers sequences. Table S3: List of miRNA assayed in pc-SEVs. Table S4: List of averaged Ct. Table S5: The amp-Ratio for each possible pair of miRNAs. Table S6: AUC values for all miRNA pairs (all patients and donors). Table S7: Prognostic potency of amp-Ratio values of miRNA pairs.
