# Supplementary material for: Diagnosis of Prostate Cancer through the Multi-Ligand Binding of Prostate-Derived Extracellular Vesicles and miRNA Analysis
Source: Life (Basel). 2023 Mar 27;13(4):885. doi: 10.3390/life13040885 (PMC10141197; doi:10.3390/life13040885)
Supplement: Supplementary file 1 [file life-13-00885-s001.zip › life-2218333-supplementary.pdf]

DIAGNOSIS OF PROSTATE CANCER VIA MULTI-LIGAND BINDING OF PROSTATE-DERIVED EXREACELLULAR VESICLES AND VESICULAR MI-RNA ANALYSIS

SUPPLEMENTARY DATA

Supplementary Table S1. Information about patients

	Age	Gleason score	Grade 1	Grade 2	Clinical stage	Risk group (1 – high, 2 – medium, 3 – low)	PSA, ng/ml	Prostate volume (sm × sm × sm × 0.52)
patient_1	68	7	3	4	T3bN0M0	1	25.0	80.0
patient_2	73	6	3	3	cT2cN0M0	1	82.0	55.0
patient_3	59	8	4	4	cT2cN0M0	1	9.7	50.0
patient_4	62	6	3	3	cT2aN0M0	3	6.5	45.0
patient_5	62	8	4	4	T3aN0M0	1	14.0	54.0
patient_6	72	7	3	4	T2cN0M0	2	5.8	40.6
patient_7	74	8	4	4	T3aN0M0	1	8.0	69.0
patient_8	71	9	4	5	T3bN1M0	1	7.0	40.0
patient_9	63	7	3	4	T2bN0M0	2	5.7	41.1
patient_10	63	7	4	3	T2aN0M0	2	16.8	120.0
patient_11	74	7	3	4	T2cN0M0	2	13.9	35.0
patient_12	66	7	3	4	T2bN0M0	2	14.5	75.0
patient_13	51	7	3	4	T2cN0M0	1	23.7	36.2
patient_14	61	6	3	3	T3aN0M0	1	6.2	50.0
patient_15	62	7	3	4	T2cN0M0	2	18.0	23.9
patient_18	49	6	3	3	T2cN0M0	2	5.8	51.0
patient_19	67	7	3	4	T3bN0M0	1	37.0	45.0
patient_20	68	7	3	4	T2cN0M0	2	13.0	33.7
patient_21	59	7	4	3	T2aN0M0	2	5.7	34.7
patient_23	74	7	4	3	T2cN0M0	1	19.0	77.0
patient_24	73	7	4	3	T2aN0M0	2	8.5	125.0
patient_25	70	8	4	4	T2bN0M0	1	4.5	38.0
patient_26	69	7	3	4	cT2aN0M0	2	7.5	42.5
patient_27	72	8	4	4	cT2cN0M0	1	10.5	66.0
patient_28	73	6	3	3	cT2aN0M0	1	9.8	34.0
patient_29	61	6	3	3	cT2bN0M0	2	7.9	37.2
patient_30	62	6	3	3	cT2bN0M0	2	12.0	35.0
patient_31	64	7	4	3	cT2cN0M0	2	8.9	40.0
patient_32	70	6	3	3	cT2cN0M0	2	7.5	33.0
patient_33	74	6	3	3	cT2cN0M0	2	15.0	160.0
patient_34	51	7	4	3	cT2cN0M0	1	27.2	18.0
patient_36	64	6	3	3	cT2aN0M0	3	6.8	41.0

patient_37	60	6	3	3	cT2aN0M0	3	4.8	26.0
patient_38	69	7	3	4	T3bN1M0	1	20.0	36.5
patient_39	65	7	3	4	T2cN0M0	1	26.0	46.8
patient_40	71	7	3	4	T2cN0M0	2	8.6	8.0

Supplementary Table S2. DNA-aptamers sequences

Aptamer	Sequence (5' → 3')	Author's names	Targets	Ref.
Apt1	acgctcggatgccactacaggttggggtcgggcatg cgtccggagaagggcaaacgagaggtca ccagcacgtccatgag	DML-7	highly metastatic PC cells (PC3, DU145)	[1]
Apt2	tgccactacagctggttcggtttggtgacttcgttcttc gttgtggtgcttagtggc	Wy-5a	PC3 cells and sections of PC	[2]
Apt3	gaattcgcgttttcgcttttgcgttttgggtcatctgctta cgatagcaatgct	Apt-PSMA	PMSA-positive SEV from urine	[3]
Ap4	catccatgggaattcgtcgaccctgcaggcatgcaa gctttccctatagtgagtcgtattactgcctaggctcga gctcg	PSMA apt-P1	SEVs secreted by LNCap cells	[4]
Apt5	cctgcaggcatgcaagctttccctatagtgagtcgtat ta	B1	PSMA on LNCap cell surface	[5]
Apt6	gcgttttcgcttttgcgttttgggtcatctgcttacgata gcaatgct	PSMA-apt	PSMA on LNCap cell surface	[6]
Apt7	ccctacggcgctaacccatgctacgaattcgttgttaa acaataggccaccgtgctacaa	AMC51	Alpha-methylacyl CoA racemase (AMACR)	[7]
Apt8	ccctacggcgctaacccagctactctagaacccatta tattttgggccaccgtgctacaa	AMC56	Alpha-methylacyl CoA racemase (AMACR)	[7]
CD63	caccccacctcgctcccgtgacactaatgcta	-	-	-
CD30	actgggcgaaacaagtctattgactatgag	C2NP	K299 cells	[8]

Apt1 (DML-7) was selected by SELEX as preferably binding highly metastatic PC cells PC-3 and DU145, but not LNCaP or 22Rv1 with low metastatic potency [1]. The Apt2 (Wy-5a) was also selected by SELEX against PC-3 cells and its specificity was confirmed using sections of metastatic PC [2]. The Apt3 was validated to bind PSMA(+) vesicles from urine [3] whereas Apt4 was shown to bind specifically to SEVs secreted by LNCap prostate cancer cells but not SEVs secreted by other types of cancer cells [4]. Apt5 was generated by SELEX against PSMA on the surface of LNCap prostate cancer cells [5]. Apt6 was used within exosome-oriented, aptamer nanoprobe-based profiling (ExoAPP) assay to quantify PSMA on the surface of vesicles secreted by different cancer cells including LNCap [6]. Apt7 and Apt8 were selected by SELEX for binding of Alpha-methylacyl CoA racemase (AMACR), also known as P504S [9], that has been proven to be highly expressed in prostate cancer cells. All sequences were retrieved from original publications and synthesized.

Supplementary Table S3. List of miRNA assayed in pc-SEVs

miRNA	ALMIR cat #	Reference
Hsa-mir-16-5p	AL16-5p	[10–12]
Hsa-mir-20a-5p	AL20a-5p	[13–15]
Hsa-mir-21-5p	AL21–5p	[11,13,16–18]
Hsa-mir-26b-5p	AL26b-5p	[19]
Hsa-mir-93-5p	AL93-5p	[14,20]
Hsa-mir-106b-5p	AL106b-5p	[21,22]
Hsa-mir-125b-5p	AL125b-5p	[10,16,23,24]
Hsa-mir-141-3p	AL141-3p	[16,18,21,23]
Hsa-mir-200b-3p	AL200b-3p	[13,21,24]
Hsa-mir-205-5p	AL205–5p	[10,24,25]
Hsa-mir-375-3p	AL375-3p	[10,14,16,18,23,24]
Hsa-mir-451a-5p	AL451a-5p	[11,26]

Supplementary Table S4. List of averaged Ct.

	Hsa-mir-16-5p	Hsa-mir-20a-5p	Hsa-mir-21-5p	Hsa-mir-26b-5p	Hsa-mir-93-5p	Hsa-mir-106b-5p	Hsa-mir-125b-5p	Hsa-mir-141-3p	Hsa-mir-200b-3p	Hsa-mir-205-5p	Hsa-mir-375-3p	Hsa-mir-451a-5p
patient_1	29.96	29.03	25.04	30.75	28.50	27.25	22.46	29.21	34.66	25.10	28.89	27.67
patient_2	30.29	28.27	25.69	29.37	28.75	28.13	22.11	28.93	39.56	25.12	29.44	26.17
patient_3	34.75	33.91	24.80	30.03	28.05	21.89	21.27	31.46	24.60	31.81	26.58	22.10
patient_4	27.46	29.12	24.97	30.13	27.29	27.56	23.21	29.87	35.45	27.51	29.83	24.02
patient_5	34.90	30.93	30.34	32.29	31.13	37.98	31.18	31.94	32.20	26.28	33.83	30.43
patient_6	29.94	30.56	29.24	32.15	29.96	33.12	30.87	31.61	32.34	26.22	31.58	26.18
patient_7	33.65	30.35	29.08	31.54	30.61	37.16	29.93	31.31	31.40	26.51	31.00	26.01
patient_8	28.30	29.85	28.01	31.84	29.13	36.11	31.38	32.24	33.22	26.26	32.64	24.94
patient_9	28.49	31.71	26.96	30.31	28.28	33.05	29.29	29.83	30.28	25.28	33.35	25.36
patient_10	27.86	28.93	25.49	30.15	26.88	33.84	27.69	29.75	30.19	28.56	33.20	24.57
patient_11	28.91	31.05	26.17	30.05	27.86	34.65	27.91	29.97	30.07	25.37	37.59	25.00
patient_12	28.17	30.84	25.27	30.36	27.01	34.04	30.35	30.38	30.46	25.68	33.58	24.15
patient_13	29.16	29.26	26.91	32.73	29.07	31.72	29.57	30.51	30.13	30.93	31.33	26.19
patient_14	30.22	30.70	27.59	35.17	30.06	31.03	29.11	30.12	30.28	30.39	34.52	27.46
patient_15	29.26	28.96	27.21	33.28	28.81	32.21	22.65	30.34	29.74	30.97	34.00	26.98
patient_18	29.21	29.15	26.81	33.57	28.44	32.84	25.80	29.91	30.10	31.20	32.69	25.44
patient_19	29.05	29.43	26.81	30.13	28.59	34.14	26.73	30.38	29.63	25.29	29.93	27.15
patient_20	31.01	30.02	29.18	30.66	30.60	32.79	29.72	30.94	31.01	25.83	33.70	27.23
patient_21	30.03	29.36	27.26	32.62	28.00	32.66	26.94	29.14	29.86	30.51	31.47	26.92
patient_23	28.39	33.38	28.43	31.49	29.08	32.41	29.45	31.25	30.63	25.69	34.50	27.06
patient_24	28.28	29.91	27.37	31.53	27.00	30.51	27.84	28.07	29.31	29.92	30.11	28.19
patient_25	30.67	29.58	27.72	30.77	28.49	31.14	29.90	30.83	30.18	25.78	34.53	24.48
patient_26	24.77	28.08	24.00	30.42	25.41	29.14	27.50	26.75	30.31	28.76	29.80	21.43
patient_27	30.16	35.08	27.58	32.12	27.81	31.61	27.16	28.77	29.52	30.31	30.50	26.51
patient_28	31.02	29.76	27.27	30.70	28.49	34.38	27.94	30.13	30.65	25.39	28.83	27.40
patient_29	28.65	30.10	26.36	31.51	29.40	32.01	29.80	30.60	33.53	25.83	28.76	25.85
patient_30	27.81	29.90	28.20	32.07	29.14	32.23	29.81	30.30	33.02	25.75	30.12	25.96
patient_31	26.52	29.60	26.44	32.11	28.44	31.10	29.97	29.85	32.67	26.32	31.01	23.29
patient_32	28.24	30.75	27.27	33.25	29.01	31.90	26.70	30.41	30.13	30.58	33.79	25.19
patient_33	29.05	30.79	27.63	33.30	29.36	32.00	26.35	30.04	30.39	30.83	34.32	25.38
patient_34	31.25	30.87	29.40	32.53	30.75	34.69	30.87	30.78	33.25	26.42	35.46	31.11
patient_36	30.16	31.04	29.43	30.95	30.36	33.18	30.63	30.58	32.42	26.30	27.16	28.01
patient_37	29.52	30.37	26.64	33.21	28.26	35.91	25.87	28.05	29.50	29.78	30.06	27.38
patient_38	26.79	29.28	25.39	30.91	28.00	31.12	30.46	30.60	31.94	26.40	31.77	24.13
patient_39	27.27	29.74	26.53	31.22	28.73	31.92	29.96	30.68	31.96	26.61	26.63	28.07
patient_40	28.45	30.80	27.72	31.00	30.04	32.54	30.39	29.86	31.97	26.25	31.95	27.61
donor_1	30.26	32.16	28.27	34.71	30.67	35.90	38.62	40.64	32.62	30.78	30.21	26.28
donor_2	30.16	31.87	27.45	33.81	29.01	34.71	37.29	35.90	31.96	30.94	31.17	26.55
donor_3	29.77	31.19	27.70	32.67	29.54	34.11	35.07	39.12	31.60	30.19	29.21	25.35
donor_4	30.18	31.70	27.58	32.99	29.50	32.70	38.20	40.81	31.86	31.03	30.03	26.25
donor_5	26.76	29.08	26.23	31.40	27.88	31.47	27.88	29.35	31.38	30.29	27.33	23.46
donor_6	26.66	28.40	25.32	29.57	28.09	26.83	26.73	29.70	28.90	30.14	27.96	24.73
donor_7	27.51	28.56	26.03	30.84	28.23	30.86	26.78	29.36	30.23	30.08	28.99	24.26
donor_8	34.26	29.86	26.63	31.82	28.32	36.77	27.83	29.48	31.13	30.28	28.92	26.03

	Hsa-mir-16-5p	Hsa-mir-20a-5p	Hsa-mir-21-5p	Hsa-mir-26b-5p	Hsa-mir-93-5p	Hsa-mir-106b-5p	Hsa-mir-125b-5p	Hsa-mir-141-3p	Hsa-mir-200b-3p	Hsa-mir-205-5p	Hsa-mir-375-3p	Hsa-mir-451a-5p
donor_9	29.10	32.64	27.03	33.92	31.01	37.57	29.25	30.13	32.28	31.05	30.03	26.78
donor_10	30.21	31.77	27.15	34.58	30.45	36.77	26.88	28.70	30.68	28.95	32.39	27.53
donor_11	29.74	31.91	27.93	34.23	29.82	37.13	28.07	28.60	31.40	29.87	28.65	26.69
donor_12	28.96	31.69	27.13	32.22	29.83	18.43	27.39	26.00	32.40	30.08	31.25	26.03
donor_13	28.21	29.76	25.53	33.95	26.45	31.98	28.39	29.71	28.35	27.81	26.51	25.52
donor_14	33.42	30.03	25.35	33.17	26.12	28.13	28.09	29.15	27.38	27.77	28.85	26.57
donor_15	28.39	29.45	25.92	31.52	26.58	27.81	27.10	28.46	27.69	27.06	26.18	30.10
donor_16	28.68	29.64	25.43	31.81	26.41	27.97	28.12	28.69	27.82	27.66	25.86	25.13
donor_17	28.90	29.00	25.98	30.54	25.31	26.61	28.06	28.36	27.11	27.64	27.57	27.27
donor_18	28.76	30.31	25.44	30.60	25.86	25.64	27.26	28.52	27.02	27.12	26.29	26.04

Supplementary Table S5. The amp-Ratio for each possible pair of miRNAs.

	mir16/mir20a	mir16/mir21	mir16/mir26b	mir16/mir93	mir16/mir106b	mir16/mir125b	mir16/mir141	mir16/mir200b	mir16/mir205	mir16/mir375	mir16/mir451a
patient_1	1.90	30.33	0.58	2.74	6.56	181.21	1.68	0.04	29.00	2.10	4.88
patient_2	4.06	24.35	1.89	2.91	4.48	290.39	2.57	0.00	36.18	1.81	17.44
patient_3	1.79	988.25	26.29	104.16	7435.39	11377.37	9.78	1134.84	7.68	288.08	6436.74
patient_4	0.32	5.64	0.16	1.12	0.93	19.12	0.19	0.00	0.97	0.19	10.85
patient_5	15.60	23.59	6.11	13.61	0.12	13.19	7.76	6.47	393.53	2.09	22.19
patient_6	0.65	1.63	0.22	0.99	0.11	0.53	0.32	0.19	13.25	0.32	13.55
patient_7	9.85	23.63	4.32	8.19	0.09	13.16	5.04	4.74	140.30	6.25	198.50
patient_8	0.34	1.22	0.09	0.56	0.00	0.12	0.07	0.03	4.13	0.05	10.26
patient_9	0.11	2.88	0.28	1.16	0.04	0.57	0.39	0.29	9.22	0.03	8.77
patient_10	0.48	5.20	0.20	1.98	0.02	1.13	0.27	0.20	0.62	0.02	9.82
patient_11	0.23	6.68	0.45	2.06	0.02	2.00	0.48	0.45	11.61	0.00	14.95
patient_12	0.16	7.46	0.22	2.24	0.02	0.22	0.22	0.20	5.63	0.02	16.30
patient_13	0.93	4.76	0.08	1.06	0.17	0.75	0.39	0.51	0.29	0.22	7.82
patient_14	0.72	6.21	0.03	1.12	0.57	2.17	1.08	0.96	0.89	0.05	6.79
patient_15	1.23	4.13	0.06	1.37	0.13	97.39	0.47	0.72	0.31	0.04	4.84
patient_18	1.04	5.28	0.05	1.71	0.08	10.67	0.62	0.54	0.25	0.09	13.64
patient_19	0.77	4.73	0.47	1.38	0.03	4.98	0.40	0.67	13.54	0.54	3.72
patient_20	1.98	3.56	1.28	1.33	0.29	2.45	1.05	1.00	36.18	0.16	13.74
patient_21	1.59	6.82	0.17	4.09	0.16	8.54	1.86	1.12	0.72	0.37	8.62
patient_23	0.03	0.98	0.12	0.62	0.06	0.48	0.14	0.21	6.52	0.01	2.52
patient_24	0.32	1.87	0.10	2.43	0.21	1.36	1.16	0.49	0.32	0.28	1.06
patient_25	2.13	7.70	0.93	4.52	0.72	1.70	0.90	1.40	29.55	0.07	72.96
patient_26	0.10	1.71	0.02	0.64	0.05	0.15	0.25	0.02	0.06	0.03	10.14
patient_27	0.03	5.98	0.26	5.10	0.37	7.98	2.63	1.55	0.90	0.79	12.51
patient_28	2.41	13.50	1.25	5.78	0.10	8.47	1.86	1.29	49.58	4.59	12.33
patient_29	0.37	4.90	0.14	0.59	0.10	0.45	0.26	0.03	7.06	0.93	6.95
patient_30	0.24	0.76	0.05	0.40	0.05	0.25	0.18	0.03	4.15	0.20	3.60
patient_31	0.12	1.06	0.02	0.26	0.04	0.09	0.10	0.01	1.14	0.04	9.33
patient_32	0.18	1.96	0.03	0.59	0.08	2.91	0.22	0.27	0.20	0.02	8.31
patient_33	0.30	2.67	0.05	0.81	0.13	6.52	0.50	0.40	0.29	0.03	12.75
patient_34	1.30	3.60	0.41	1.41	0.09	1.29	1.38	0.25	28.40	0.05	1.10
patient_36	0.54	1.67	0.58	0.87	0.12	0.72	0.75	0.21	14.53	8.04	4.46
patient_37	0.56	7.38	0.08	2.39	0.01	12.57	2.77	1.01	0.84	0.69	4.42
patient_38	0.18	2.64	0.06	0.43	0.05	0.08	0.07	0.03	1.31	0.03	6.36
patient_39	0.18	1.68	0.06	0.36	0.04	0.16	0.09	0.04	1.58	1.56	0.58
patient_40	0.20	1.66	0.17	0.33	0.06	0.26	0.38	0.09	4.58	0.09	1.79

	mir20a/mir16	mir20a/mir21	mir20a/mir26b	mir20a/mir93	mir20a/mir106b	mir20a/mir125b	mir20a/mir141	mir20a/mir200b	mir20a/mir205	mir20a/mir375	mir20a/mir451a
patient_1	0.53	15.98	0.30	1.45	3.45	95.49	0.88	0.02	15.28	1.11	2.57
patient_2	0.25	6.00	0.47	0.72	1.10	71.60	0.63	0.00	8.92	0.45	4.30
patient_3	0.56	553.49	14.73	58.34	4164.33	6372.11	5.48	635.59	4.30	161.34	3605.02
patient_4	3.16	17.82	0.50	3.55	2.94	60.40	0.60	0.01	3.06	0.61	34.29
patient_5	0.06	1.51	0.39	0.87	0.01	0.85	0.50	0.41	25.23	0.13	1.42
patient_6	1.53	2.50	0.33	1.52	0.17	0.81	0.48	0.29	20.34	0.49	20.79
patient_7	0.10	2.40	0.44	0.83	0.01	1.34	0.51	0.48	14.25	0.63	20.16
patient_8	2.92	3.58	0.25	1.64	0.01	0.35	0.19	0.10	12.07	0.14	30.00
patient_9	9.35	26.90	2.64	10.81	0.40	5.35	3.68	2.70	86.22	0.32	81.99
patient_10	2.10	10.92	0.43	4.16	0.03	2.37	0.57	0.42	1.30	0.05	20.64
patient_11	4.42	29.53	2.00	9.12	0.08	8.84	2.12	1.97	51.31	0.01	66.09
patient_12	6.34	47.27	1.39	14.22	0.11	1.40	1.37	1.30	35.69	0.15	103.29
patient_13	1.07	5.10	0.09	1.14	0.18	0.80	0.42	0.55	0.31	0.24	8.38
patient_14	1.39	8.61	0.05	1.55	0.79	3.01	1.49	1.34	1.23	0.07	9.41
patient_15	0.81	3.37	0.05	1.12	0.11	79.30	0.38	0.58	0.25	0.03	3.94
patient_18	0.96	5.05	0.05	1.64	0.08	10.22	0.59	0.52	0.24	0.09	13.07
patient_19	1.31	6.17	0.62	1.80	0.04	6.50	0.52	0.87	17.67	0.71	4.86
patient_20	0.50	1.79	0.64	0.67	0.15	1.24	0.53	0.50	18.24	0.08	6.93
patient_21	0.63	4.29	0.10	2.58	0.10	5.38	1.17	0.71	0.45	0.23	5.43
patient_23	31.72	30.97	3.70	19.71	1.96	15.24	4.38	6.73	206.85	0.46	80.00
patient_24	3.10	5.82	0.33	7.53	0.66	4.21	3.59	1.51	1.00	0.87	3.31
patient_25	0.47	3.62	0.44	2.12	0.34	0.80	0.42	0.66	13.88	0.03	34.28
patient_26	9.90	16.90	0.20	6.35	0.48	1.50	2.51	0.21	0.62	0.30	100.39
patient_27	30.24	180.80	7.79	154.14	11.07	241.36	79.49	47.00	27.32	23.94	378.36
patient_28	0.42	5.61	0.52	2.40	0.04	3.52	0.77	0.54	20.61	1.91	5.13
patient_29	2.72	13.34	0.37	1.62	0.27	1.23	0.70	0.09	19.24	2.52	18.92
patient_30	4.25	3.23	0.22	1.69	0.20	1.06	0.75	0.11	17.65	0.85	15.30
patient_31	8.46	8.94	0.18	2.23	0.35	0.77	0.84	0.12	9.67	0.38	78.99
patient_32	5.71	11.21	0.18	3.35	0.45	16.59	1.27	1.54	1.13	0.12	47.42
patient_33	3.33	8.90	0.18	2.68	0.43	21.72	1.68	1.32	0.97	0.09	42.46
patient_34	0.77	2.77	0.32	1.09	0.07	1.00	1.06	0.19	21.89	0.04	0.85
patient_36	1.84	3.07	1.07	1.61	0.23	1.33	1.38	0.38	26.73	14.80	8.21
patient_37	1.80	13.27	0.14	4.30	0.02	22.61	4.99	1.83	1.50	1.24	7.95
patient_38	5.60	14.80	0.32	2.42	0.28	0.44	0.40	0.16	7.34	0.18	35.61
patient_39	5.54	9.30	0.36	2.02	0.22	0.86	0.52	0.22	8.78	8.62	3.20
patient_40	5.12	8.49	0.87	1.69	0.30	1.33	1.93	0.45	23.46	0.45	9.17

	mir21/mir16	mir21/mir20a	mir21/mir26b	mir21/mir93	mir21/mir106b	mir21/mir125b	mir21/mir141	mir21/mir200b	mir21/mir205	mir21/mir375	mir21/mir451a
patient_1	0.03	0.06	0.02	0.09	0.22	5.98	0.06	0.00	0.96	0.07	0.16
patient_2	0.04	0.17	0.08	0.12	0.18	11.93	0.11	0.00	1.49	0.07	0.72
patient_3	0.00	0.00	0.03	0.11	7.52	11.51	0.01	1.15	0.01	0.29	6.51
patient_4	0.18	0.06	0.03	0.20	0.17	3.39	0.03	0.00	0.17	0.03	1.92
patient_5	0.04	0.66	0.26	0.58	0.01	0.56	0.33	0.27	16.68	0.09	0.94
patient_6	0.61	0.40	0.13	0.61	0.07	0.32	0.19	0.12	8.15	0.20	8.33
patient_7	0.04	0.42	0.18	0.35	0.00	0.56	0.21	0.20	5.94	0.26	8.40
patient_8	0.82	0.28	0.07	0.46	0.00	0.10	0.05	0.03	3.37	0.04	8.38
patient_9	0.35	0.04	0.10	0.40	0.01	0.20	0.14	0.10	3.20	0.01	3.05
patient_10	0.19	0.09	0.04	0.38	0.00	0.22	0.05	0.04	0.12	0.00	1.89
patient_11	0.15	0.03	0.07	0.31	0.00	0.30	0.07	0.07	1.74	0.00	2.24
patient_12	0.13	0.02	0.03	0.30	0.00	0.03	0.03	0.03	0.76	0.00	2.19
patient_13	0.21	0.20	0.02	0.22	0.04	0.16	0.08	0.11	0.06	0.05	1.64
patient_14	0.16	0.12	0.01	0.18	0.09	0.35	0.17	0.16	0.14	0.01	1.09
patient_15	0.24	0.30	0.01	0.33	0.03	23.56	0.11	0.17	0.07	0.01	1.17
patient_18	0.19	0.20	0.01	0.32	0.02	2.02	0.12	0.10	0.05	0.02	2.59
patient_19	0.21	0.16	0.10	0.29	0.01	1.05	0.08	0.14	2.86	0.11	0.79
patient_20	0.28	0.56	0.36	0.37	0.08	0.69	0.29	0.28	10.18	0.04	3.87
patient_21	0.15	0.23	0.02	0.60	0.02	1.25	0.27	0.16	0.11	0.05	1.26
patient_23	1.02	0.03	0.12	0.64	0.06	0.49	0.14	0.22	6.68	0.01	2.58
patient_24	0.53	0.17	0.06	1.29	0.11	0.72	0.62	0.26	0.17	0.15	0.57
patient_25	0.13	0.28	0.12	0.59	0.09	0.22	0.12	0.18	3.84	0.01	9.47
patient_26	0.59	0.06	0.01	0.38	0.03	0.09	0.15	0.01	0.04	0.02	5.94
patient_27	0.17	0.01	0.04	0.85	0.06	1.33	0.44	0.26	0.15	0.13	2.09
patient_28	0.07	0.18	0.09	0.43	0.01	0.63	0.14	0.10	3.67	0.34	0.91
patient_29	0.20	0.07	0.03	0.12	0.02	0.09	0.05	0.01	1.44	0.19	1.42
patient_30	1.31	0.31	0.07	0.52	0.06	0.33	0.23	0.04	5.46	0.26	4.73
patient_31	0.95	0.11	0.02	0.25	0.04	0.09	0.09	0.01	1.08	0.04	8.83
patient_32	0.51	0.09	0.02	0.30	0.04	1.48	0.11	0.14	0.10	0.01	4.23
patient_33	0.37	0.11	0.02	0.30	0.05	2.44	0.19	0.15	0.11	0.01	4.77
patient_34	0.28	0.36	0.11	0.39	0.03	0.36	0.38	0.07	7.89	0.01	0.31
patient_36	0.60	0.33	0.35	0.52	0.07	0.43	0.45	0.13	8.72	4.83	2.68
patient_37	0.14	0.08	0.01	0.32	0.00	1.70	0.38	0.14	0.11	0.09	0.60
patient_38	0.38	0.07	0.02	0.16	0.02	0.03	0.03	0.01	0.50	0.01	2.41
patient_39	0.60	0.11	0.04	0.22	0.02	0.09	0.06	0.02	0.94	0.93	0.34
patient_40	0.60	0.12	0.10	0.20	0.04	0.16	0.23	0.05	2.76	0.05	1.08

	mir26b/mir16	mir26b/mir20a	mir26b/mir21	mir26b/mir93	mir26b/mir106b	mir26b/mir125b	mir26b/mir141	mir26b/mir200b	mir26b/mir205	mir26b/mir375	mir26b/mir451a
patient_1	1.73	3.28	52.43	4.74	11.33	313.26	2.90	0.07	50.13	3.63	8.44
patient_2	0.53	2.14	12.86	1.54	2.36	153.36	1.35	0.00	19.11	0.96	9.21
patient_3	0.04	0.07	37.58	3.96	282.77	432.68	0.37	43.16	0.29	10.96	244.79
patient_4	6.34	2.01	35.79	7.13	5.91	121.26	1.20	0.02	6.15	1.23	68.85
patient_5	0.16	2.55	3.86	2.23	0.02	2.16	1.27	1.06	64.40	0.34	3.63
patient_6	4.62	3.01	7.51	4.57	0.51	2.43	1.46	0.88	61.19	1.49	62.56
patient_7	0.23	2.28	5.48	1.90	0.02	3.05	1.17	1.10	32.51	1.45	46.00
patient_8	11.58	3.96	14.18	6.51	0.05	1.37	0.76	0.38	47.81	0.57	118.81
patient_9	3.54	0.38	10.20	4.10	0.15	2.03	1.39	1.02	32.68	0.12	31.08
patient_10	4.88	2.32	25.37	9.67	0.08	5.51	1.32	0.97	3.01	0.12	47.93
patient_11	2.21	0.50	14.79	4.57	0.04	4.43	1.06	0.99	25.70	0.01	33.11
patient_12	4.56	0.72	34.03	10.24	0.08	1.01	0.99	0.93	25.70	0.11	74.36
patient_13	11.93	11.12	56.73	12.70	2.01	8.95	4.65	6.07	3.47	2.65	93.22
patient_14	30.79	22.21	191.17	34.43	17.62	66.78	33.15	29.70	27.42	1.56	209.07
patient_15	16.23	19.93	67.08	22.24	2.10	1580.38	7.66	11.66	4.95	0.61	78.62
patient_18	20.46	21.36	107.95	34.97	1.65	218.29	12.60	11.09	5.17	1.84	279.20
patient_19	2.11	1.62	9.98	2.90	0.06	10.50	0.84	1.41	28.56	1.15	7.85
patient_20	0.78	1.55	2.78	1.04	0.23	1.92	0.82	0.78	28.30	0.12	10.75
patient_21	6.03	9.57	41.08	24.65	0.98	51.45	11.19	6.78	4.33	2.22	51.93
patient_23	8.57	0.27	8.37	5.32	0.53	4.12	1.18	1.82	55.87	0.12	21.61
patient_24	9.55	3.08	17.91	23.18	2.03	12.95	11.04	4.65	3.06	2.69	10.17
patient_25	1.08	2.29	8.30	4.87	0.78	1.83	0.96	1.51	31.83	0.07	78.59
patient_26	50.03	5.05	85.39	32.07	2.43	7.56	12.68	1.08	3.15	1.53	507.28
patient_27	3.88	0.13	23.22	19.80	1.42	31.00	10.21	6.04	3.51	3.08	48.60
patient_28	0.80	1.93	10.80	4.63	0.08	6.78	1.49	1.03	39.68	3.68	9.87
patient_29	7.28	2.67	35.62	4.32	0.71	3.28	1.88	0.25	51.38	6.74	50.55
patient_30	19.16	4.51	14.59	7.62	0.89	4.78	3.40	0.52	79.58	3.86	68.98
patient_31	48.30	5.71	51.03	12.72	2.01	4.40	4.79	0.68	55.16	2.14	450.78
patient_32	32.11	5.63	63.06	18.83	2.53	93.39	7.14	8.68	6.36	0.68	266.88
patient_33	19.01	5.71	50.83	15.31	2.47	124.00	9.56	7.55	5.53	0.49	242.36
patient_34	2.43	3.16	8.76	3.43	0.22	3.15	3.36	0.61	69.08	0.13	2.68
patient_36	1.73	0.94	2.88	1.51	0.21	1.25	1.29	0.36	25.09	13.89	7.71
patient_37	12.91	7.18	95.26	30.86	0.15	162.33	35.80	13.10	10.79	8.87	57.09
patient_38	17.34	3.10	45.84	7.50	0.86	1.37	1.24	0.49	22.74	0.55	110.27
patient_39	15.48	2.79	25.98	5.64	0.62	2.40	1.45	0.60	24.53	24.09	8.94
patient_40	5.87	1.15	9.73	1.94	0.34	1.53	2.21	0.51	26.88	0.52	10.51

	mir93/mir16	mir93/mir20a	mir93/mir21	mir93/mir26b	mir93/mir106b	mir93/mir125b	mir93/mir141	mir93/mir200b	mir93/mir205	mir93/mir375	mir93/mir451a
patient_1	0.36	0.69	11.05	0.21	2.39	66.05	0.61	0.01	10.57	0.77	1.78
patient_2	0.34	1.39	8.37	0.65	1.54	99.82	0.88	0.00	12.44	0.62	6.00
patient_3	0.01	0.02	9.49	0.25	71.39	109.23	0.09	10.90	0.07	2.77	61.80
patient_4	0.89	0.28	5.02	0.14	0.83	17.01	0.17	0.00	0.86	0.17	9.66
patient_5	0.07	1.15	1.73	0.45	0.01	0.97	0.57	0.48	28.91	0.15	1.63
patient_6	1.01	0.66	1.64	0.22	0.11	0.53	0.32	0.19	13.39	0.33	13.69
patient_7	0.12	1.20	2.88	0.53	0.01	1.61	0.61	0.58	17.12	0.76	24.23
patient_8	1.78	0.61	2.18	0.15	0.01	0.21	0.12	0.06	7.34	0.09	18.25
patient_9	0.86	0.09	2.49	0.24	0.04	0.50	0.34	0.25	7.97	0.03	7.58
patient_10	0.50	0.24	2.62	0.10	0.01	0.57	0.14	0.10	0.31	0.01	4.96
patient_11	0.48	0.11	3.24	0.22	0.01	0.97	0.23	0.22	5.62	0.00	7.25
patient_12	0.45	0.07	3.32	0.10	0.01	0.10	0.10	0.09	2.51	0.01	7.26
patient_13	0.94	0.88	4.47	0.08	0.16	0.71	0.37	0.48	0.27	0.21	7.34
patient_14	0.89	0.65	5.55	0.03	0.51	1.94	0.96	0.86	0.80	0.05	6.07
patient_15	0.73	0.90	3.02	0.04	0.09	71.07	0.34	0.52	0.22	0.03	3.54
patient_18	0.59	0.61	3.09	0.03	0.05	6.24	0.36	0.32	0.15	0.05	7.98
patient_19	0.73	0.56	3.44	0.34	0.02	3.62	0.29	0.49	9.84	0.39	2.71
patient_20	0.75	1.49	2.67	0.96	0.22	1.84	0.79	0.75	27.21	0.12	10.33
patient_21	0.24	0.39	1.67	0.04	0.04	2.09	0.45	0.27	0.18	0.09	2.11
patient_23	1.61	0.05	1.57	0.19	0.10	0.77	0.22	0.34	10.49	0.02	4.06
patient_24	0.41	0.13	0.77	0.04	0.09	0.56	0.48	0.20	0.13	0.12	0.44
patient_25	0.22	0.47	1.70	0.21	0.16	0.38	0.20	0.31	6.54	0.02	16.14
patient_26	1.56	0.16	2.66	0.03	0.08	0.24	0.40	0.03	0.10	0.05	15.82
patient_27	0.20	0.01	1.17	0.05	0.07	1.57	0.52	0.30	0.18	0.16	2.45
patient_28	0.17	0.42	2.33	0.22	0.02	1.46	0.32	0.22	8.57	0.79	2.13
patient_29	1.68	0.62	8.24	0.23	0.16	0.76	0.44	0.06	11.89	1.56	11.70
patient_30	2.52	0.59	1.92	0.13	0.12	0.63	0.45	0.07	10.45	0.51	9.06
patient_31	3.80	0.45	4.01	0.08	0.16	0.35	0.38	0.05	4.34	0.17	35.45
patient_32	1.71	0.30	3.35	0.05	0.13	4.96	0.38	0.46	0.34	0.04	14.18
patient_33	1.24	0.37	3.32	0.07	0.16	8.10	0.62	0.49	0.36	0.03	15.83
patient_34	0.71	0.92	2.55	0.29	0.07	0.92	0.98	0.18	20.16	0.04	0.78
patient_36	1.14	0.62	1.91	0.66	0.14	0.83	0.86	0.24	16.61	9.20	5.10
patient_37	0.42	0.23	3.09	0.03	0.01	5.26	1.16	0.42	0.35	0.29	1.85
patient_38	2.31	0.41	6.11	0.13	0.11	0.18	0.17	0.07	3.03	0.07	14.71
patient_39	2.75	0.50	4.61	0.18	0.11	0.43	0.26	0.11	4.35	4.27	1.59
patient_40	3.02	0.59	5.01	0.52	0.18	0.79	1.14	0.26	13.85	0.27	5.42

	mir106b/mir16	mir106b/mir20a	mir106b/mir21	mir106b/mir26b	mir106b/mir93	mir106b/mir125b	mir106b/mir141	mir106b/mir200b	mir106b/mir205	mir106b/mir375	mir106b/mir451a
patient_1	0.15	0.29	4.63	0.09	0.42	27.64	0.26	0.01	4.42	0.32	0.74
patient_2	0.22	0.91	5.44	0.42	0.65	64.86	0.57	0.00	8.08	0.40	3.90
patient_3	0.00	0.00	0.13	0.00	0.01	1.53	0.00	0.15	0.00	0.04	0.87
patient_4	1.07	0.34	6.05	0.17	1.21	20.51	0.20	0.00	1.04	0.21	11.65
patient_5	8.46	131.92	199.52	51.67	115.11	111.52	65.60	54.72	3327.72	17.67	187.62
patient_6	9.02	5.88	14.67	1.95	8.93	4.74	2.84	1.72	119.53	2.90	122.21
patient_7	11.43	112.59	270.21	49.34	93.68	150.41	57.60	54.23	1604.08	71.47	2269.49
patient_8	224.06	76.62	274.30	19.35	125.98	26.56	14.65	7.43	924.87	11.07	2298.57
patient_9	23.62	2.53	67.97	6.67	27.32	13.53	9.29	6.81	217.84	0.81	207.16
patient_10	63.08	30.03	327.92	12.93	125.04	71.22	17.06	12.56	38.89	1.56	619.63
patient_11	53.67	12.14	358.61	24.24	110.76	107.36	25.74	23.94	623.00	0.13	802.51
patient_12	58.28	9.20	434.77	12.77	130.81	12.86	12.61	11.93	328.29	1.37	949.98
patient_13	5.93	5.53	28.22	0.50	6.32	4.45	2.31	3.02	1.73	1.32	46.38
patient_14	1.75	1.26	10.85	0.06	1.95	3.79	1.88	1.69	1.56	0.09	11.86
patient_15	7.74	9.50	31.99	0.48	10.60	753.64	3.65	5.56	2.36	0.29	37.49
patient_18	12.38	12.92	65.31	0.60	21.16	132.06	7.62	6.71	3.13	1.11	168.91
patient_19	33.97	26.03	160.68	16.10	46.74	169.12	13.49	22.67	460.02	18.46	126.47
patient_20	3.43	6.80	12.18	4.38	4.56	8.40	3.59	3.42	123.98	0.53	47.09
patient_21	6.17	9.80	42.08	1.02	25.25	52.69	11.46	6.94	4.44	2.27	53.19
patient_23	16.20	0.51	15.82	1.89	10.07	7.79	2.24	3.44	105.67	0.23	40.87
patient_24	4.71	1.52	8.83	0.49	11.43	6.39	5.44	2.29	1.51	1.32	5.02
patient_25	1.39	2.95	10.69	1.29	6.27	2.36	1.24	1.94	41.02	0.10	101.29
patient_26	20.59	2.08	35.14	0.41	13.20	3.11	5.22	0.44	1.30	0.63	208.77
patient_27	2.73	0.09	16.33	0.70	13.92	21.80	7.18	4.25	2.47	2.16	34.18
patient_28	10.25	24.66	138.33	12.81	59.28	86.84	19.06	13.25	508.18	47.09	126.44
patient_29	10.26	3.77	50.22	1.41	6.09	4.62	2.65	0.35	72.44	9.50	71.26
patient_30	21.42	5.04	16.30	1.12	8.51	5.34	3.80	0.58	88.93	4.31	77.08
patient_31	24.07	2.84	25.43	0.50	6.34	2.19	2.39	0.34	27.49	1.07	224.62
patient_32	12.67	2.22	24.89	0.39	7.43	36.86	2.82	3.43	2.51	0.27	105.34
patient_33	7.69	2.31	20.57	0.40	6.20	50.18	3.87	3.05	2.24	0.20	98.07
patient_34	10.90	14.14	39.25	4.48	15.36	14.12	15.06	2.72	309.66	0.59	12.03
patient_36	8.07	4.39	13.45	4.67	7.06	5.84	6.04	1.69	117.27	64.92	36.02
patient_37	83.51	46.44	616.20	6.47	199.60	1050.08	231.59	84.76	69.82	57.39	369.33
patient_38	20.11	3.59	53.16	1.16	8.70	1.59	1.44	0.57	26.37	0.64	127.88
patient_39	25.02	4.51	41.98	1.62	9.11	3.88	2.35	0.97	39.63	38.92	14.44
patient_40	17.01	3.32	28.21	2.90	5.63	4.43	6.40	1.48	77.95	1.50	30.47

	mir125b/mir16	mir125b/mir20a	mir125b/mir21	mir125b/mir26b	mir125b/mir93	mir125b/mir106b	mir125b/mir141	mir125b/mir200b	mir125b/mir205	mir125b/mir375	mir125b/mir451a
patient_1	0.01	0.01	0.17	0.00	0.02	0.04	0.01	0.00	0.16	0.01	0.03
patient_2	0.00	0.01	0.08	0.01	0.01	0.02	0.01	0.00	0.12	0.01	0.06
patient_3	0.00	0.00	0.09	0.00	0.01	0.65	0.00	0.10	0.00	0.03	0.57
patient_4	0.05	0.02	0.30	0.01	0.06	0.05	0.01	0.00	0.05	0.01	0.57
patient_5	0.08	1.18	1.79	0.46	1.03	0.01	0.59	0.49	29.84	0.16	1.68
patient_6	1.90	1.24	3.10	0.41	1.88	0.21	0.60	0.36	25.22	0.61	25.78
patient_7	0.08	0.75	1.80	0.33	0.62	0.01	0.38	0.36	10.66	0.48	15.09
patient_8	8.44	2.88	10.33	0.73	4.74	0.04	0.55	0.28	34.82	0.42	86.54
patient_9	1.75	0.19	5.02	0.49	2.02	0.07	0.69	0.50	16.10	0.06	15.31
patient_10	0.89	0.42	4.60	0.18	1.76	0.01	0.24	0.18	0.55	0.02	8.70
patient_11	0.50	0.11	3.34	0.23	1.03	0.01	0.24	0.22	5.80	0.00	7.48
patient_12	4.53	0.72	33.81	0.99	10.17	0.08	0.98	0.93	25.53	0.11	73.88
patient_13	1.33	1.24	6.34	0.11	1.42	0.22	0.52	0.68	0.39	0.30	10.41
patient_14	0.46	0.33	2.86	0.01	0.52	0.26	0.50	0.44	0.41	0.02	3.13
patient_15	0.01	0.01	0.04	0.00	0.01	0.00	0.00	0.01	0.00	0.00	0.05
patient_18	0.09	0.10	0.49	0.00	0.16	0.01	0.06	0.05	0.02	0.01	1.28
patient_19	0.20	0.15	0.95	0.10	0.28	0.01	0.08	0.13	2.72	0.11	0.75
patient_20	0.41	0.81	1.45	0.52	0.54	0.12	0.43	0.41	14.76	0.06	5.61
patient_21	0.12	0.19	0.80	0.02	0.48	0.02	0.22	0.13	0.08	0.04	1.01
patient_23	2.08	0.07	2.03	0.24	1.29	0.13	0.29	0.44	13.57	0.03	5.25
patient_24	0.74	0.24	1.38	0.08	1.79	0.16	0.85	0.36	0.24	0.21	0.79
patient_25	0.59	1.25	4.54	0.55	2.66	0.42	0.53	0.82	17.40	0.04	42.97
patient_26	6.62	0.67	11.30	0.13	4.24	0.32	1.68	0.14	0.42	0.20	67.13
patient_27	0.13	0.00	0.75	0.03	0.64	0.05	0.33	0.19	0.11	0.10	1.57
patient_28	0.12	0.28	1.59	0.15	0.68	0.01	0.22	0.15	5.85	0.54	1.46
patient_29	2.22	0.81	10.87	0.31	1.32	0.22	0.57	0.08	15.67	2.06	15.42
patient_30	4.01	0.94	3.05	0.21	1.59	0.19	0.71	0.11	16.66	0.81	14.44
patient_31	10.99	1.30	11.61	0.23	2.89	0.46	1.09	0.15	12.55	0.49	102.56
patient_32	0.34	0.06	0.68	0.01	0.20	0.03	0.08	0.09	0.07	0.01	2.86
patient_33	0.15	0.05	0.41	0.01	0.12	0.02	0.08	0.06	0.04	0.00	1.95
patient_34	0.77	1.00	2.78	0.32	1.09	0.07	1.07	0.19	21.93	0.04	0.85
patient_36	1.38	0.75	2.30	0.80	1.21	0.17	1.03	0.29	20.07	11.11	6.16
patient_37	0.08	0.04	0.59	0.01	0.19	0.00	0.22	0.08	0.07	0.05	0.35
patient_38	12.67	2.26	33.49	0.73	5.48	0.63	0.91	0.36	16.61	0.40	80.57
patient_39	6.44	1.16	10.81	0.42	2.34	0.26	0.61	0.25	10.20	10.02	3.72
patient_40	3.84	0.75	6.37	0.65	1.27	0.23	1.45	0.33	17.60	0.34	6.88

	mir141/mir16	mir141/mir20a	mir141/mir21	mir141/mir26b	mir141/mir93	mir141/mir106b	mir141/mir125b	mir141/mir200b	mir141/mir205	mir141/mir375	mir141/mir451a
patient_1	0.60	1.13	18.08	0.34	1.64	3.91	108.02	0.02	17.29	1.25	2.91
patient_2	0.39	1.58	9.49	0.74	1.13	1.75	113.21	0.00	14.10	0.71	6.80
patient_3	0.10	0.18	101.01	2.69	10.65	759.97	1162.87	115.99	0.78	29.44	657.90
patient_4	5.29	1.67	29.85	0.83	5.95	4.93	101.15	0.02	5.13	1.02	57.43
patient_5	0.13	2.01	3.04	0.79	1.75	0.02	1.70	0.83	50.73	0.27	2.86
patient_6	3.17	2.07	5.16	0.69	3.14	0.35	1.67	0.60	42.03	1.02	42.97
patient_7	0.20	1.95	4.69	0.86	1.63	0.02	2.61	0.94	27.85	1.24	39.40
patient_8	15.30	5.23	18.73	1.32	8.60	0.07	1.81	0.51	63.15	0.76	156.95
patient_9	2.54	0.27	7.32	0.72	2.94	0.11	1.46	0.73	23.46	0.09	22.31
patient_10	3.70	1.76	19.22	0.76	7.33	0.06	4.17	0.74	2.28	0.09	36.32
patient_11	2.08	0.47	13.93	0.94	4.30	0.04	4.17	0.93	24.20	0.01	31.17
patient_12	4.62	0.73	34.47	1.01	10.37	0.08	1.02	0.95	26.03	0.11	75.32
patient_13	2.56	2.39	12.20	0.22	2.73	0.43	1.93	1.31	0.75	0.57	20.05
patient_14	0.93	0.67	5.77	0.03	1.04	0.53	2.01	0.90	0.83	0.05	6.31
patient_15	2.12	2.60	8.76	0.13	2.90	0.27	206.36	1.52	0.65	0.08	10.27
patient_18	1.62	1.70	8.57	0.08	2.78	0.13	17.32	0.88	0.41	0.15	22.16
patient_19	2.52	1.93	11.91	1.19	3.46	0.07	12.53	1.68	34.10	1.37	9.37
patient_20	0.95	1.89	3.39	1.22	1.27	0.28	2.34	0.95	34.54	0.15	13.12
patient_21	0.54	0.86	3.67	0.09	2.20	0.09	4.60	0.61	0.39	0.20	4.64
patient_23	7.24	0.23	7.07	0.84	4.50	0.45	3.48	1.54	47.19	0.10	18.25
patient_24	0.87	0.28	1.62	0.09	2.10	0.18	1.17	0.42	0.28	0.24	0.92
patient_25	1.12	2.38	8.60	1.04	5.05	0.80	1.90	1.56	33.00	0.08	81.48
patient_26	3.95	0.40	6.73	0.08	2.53	0.19	0.60	0.09	0.25	0.12	40.01
patient_27	0.38	0.01	2.27	0.10	1.94	0.14	3.04	0.59	0.34	0.30	4.76
patient_28	0.54	1.29	7.26	0.67	3.11	0.05	4.56	0.70	26.66	2.47	6.63
patient_29	3.87	1.42	18.93	0.53	2.30	0.38	1.74	0.13	27.31	3.58	26.86
patient_30	5.64	1.33	4.29	0.29	2.24	0.26	1.41	0.15	23.43	1.13	20.31
patient_31	10.08	1.19	10.65	0.21	2.65	0.42	0.92	0.14	11.51	0.45	94.10
patient_32	4.50	0.79	8.83	0.14	2.64	0.35	13.08	1.22	0.89	0.10	37.39
patient_33	1.99	0.60	5.31	0.10	1.60	0.26	12.96	0.79	0.58	0.05	25.34
patient_34	0.72	0.94	2.61	0.30	1.02	0.07	0.94	0.18	20.56	0.04	0.80
patient_36	1.34	0.73	2.22	0.77	1.17	0.17	0.97	0.28	19.40	10.74	5.96
patient_37	0.36	0.20	2.66	0.03	0.86	0.00	4.53	0.37	0.30	0.25	1.59
patient_38	13.96	2.49	36.91	0.81	6.04	0.69	1.10	0.39	18.31	0.44	88.80
patient_39	10.64	1.92	17.86	0.69	3.87	0.43	1.65	0.41	16.86	16.56	6.14
patient_40	2.66	0.52	4.41	0.45	0.88	0.16	0.69	0.23	12.17	0.23	4.76

	mir200b/mir16	mir200b/mir20a	mir200b/mir21	mir200b/mir26b	mir200b/mir93	mir200b/mir106b	mir200b/mir125b	mir200b/mir141	mir200b/mir205	mir200b/mir375
patient_1	25.99	49.33	788.28	15.04	71.31	170.40	4710.14	43.60	753.81	54.56
patient_2	617.31	2503.62	15029.98	1168.89	1795.74	2763.59	179257.03	1583.47	22334.28	1117.49
patient_3	0.00	0.00	0.87	0.02	0.09	6.55	10.03	0.01	0.01	0.25
patient_4	254.20	80.46	1434.04	40.07	285.73	236.90	4859.44	48.04	246.29	49.24
patient_5	0.15	2.41	3.65	0.94	2.10	0.02	2.04	1.20	60.81	0.32
patient_6	5.25	3.42	8.54	1.14	5.20	0.58	2.76	1.66	69.59	1.69
patient_7	0.21	2.08	4.98	0.91	1.73	0.02	2.77	1.06	29.58	1.32
patient_8	30.14	10.31	36.90	2.60	16.95	0.13	3.57	1.97	124.40	1.49
patient_9	3.47	0.37	9.98	0.98	4.01	0.15	1.99	1.36	31.97	0.12
patient_10	5.02	2.39	26.11	1.03	9.95	0.08	5.67	1.36	3.10	0.12
patient_11	2.24	0.51	14.98	1.01	4.63	0.04	4.48	1.08	26.02	0.01
patient_12	4.89	0.77	36.45	1.07	10.97	0.08	1.08	1.06	27.52	0.11
patient_13	1.96	1.83	9.34	0.16	2.09	0.33	1.47	0.77	0.57	0.44
patient_14	1.04	0.75	6.44	0.03	1.16	0.59	2.25	1.12	0.92	0.05
patient_15	1.39	1.71	5.75	0.09	1.91	0.18	135.57	0.66	0.42	0.05
patient_18	1.85	1.93	9.73	0.09	3.15	0.15	19.68	1.14	0.47	0.17
patient_19	1.50	1.15	7.09	0.71	2.06	0.04	7.46	0.60	20.29	0.81
patient_20	1.00	1.99	3.56	1.28	1.33	0.29	2.45	1.05	36.21	0.16
patient_21	0.89	1.41	6.06	0.15	3.64	0.14	7.59	1.65	0.64	0.33
patient_23	4.71	0.15	4.60	0.55	2.93	0.29	2.26	0.65	30.73	0.07
patient_24	2.05	0.66	3.85	0.21	4.98	0.44	2.78	2.37	0.66	0.58
patient_25	0.72	1.52	5.51	0.66	3.23	0.52	1.22	0.64	21.15	0.05
patient_26	46.40	4.69	79.20	0.93	29.75	2.25	7.01	11.76	2.92	1.42
patient_27	0.64	0.02	3.85	0.17	3.28	0.24	5.13	1.69	0.58	0.51
patient_28	0.77	1.86	10.44	0.97	4.47	0.08	6.55	1.44	38.35	3.55
patient_29	29.42	10.80	144.03	4.04	17.47	2.87	13.25	7.61	207.75	27.24
patient_30	36.96	8.70	28.13	1.93	14.69	1.73	9.21	6.55	153.46	7.44
patient_31	71.07	8.40	75.08	1.47	18.71	2.95	6.47	7.05	81.16	3.15
patient_32	3.70	0.65	7.26	0.12	2.17	0.29	10.76	0.82	0.73	0.08
patient_33	2.52	0.76	6.73	0.13	2.03	0.33	16.43	1.27	0.73	0.07
patient_34	4.01	5.21	14.45	1.65	5.66	0.37	5.20	5.54	114.00	0.22
patient_36	4.78	2.60	7.96	2.77	4.18	0.59	3.46	3.58	69.45	38.44
patient_37	0.99	0.55	7.27	0.08	2.36	0.01	12.39	2.73	0.82	0.68
patient_38	35.43	6.33	93.67	2.04	15.32	1.76	2.80	2.54	46.46	1.12
patient_39	25.73	4.64	43.18	1.66	9.37	1.03	4.00	2.42	40.76	40.03
patient_40	11.48	2.24	19.03	1.96	3.80	0.67	2.99	4.32	52.60	1.01

	mir205/mir16	mir205/mir20a	mir205/mir21	mir205/mir26b	mir205/mir93	mir205/mir106b	mir205/mir125b	mir205/mir141	mir205/mir200b	mir205/mir375	mir205/mir451a
patient_1	0.03	0.07	1.05	0.02	0.09	0.23	6.25	0.06	0.00	0.07	0.17
patient_2	0.03	0.11	0.67	0.05	0.08	0.12	8.03	0.07	0.00	0.05	0.48
patient_3	0.13	0.23	128.74	3.43	13.57	968.63	1482.16	1.27	147.84	37.53	838.53
patient_4	1.03	0.33	5.82	0.16	1.16	0.96	19.73	0.20	0.00	0.20	11.20
patient_5	0.00	0.04	0.06	0.02	0.03	0.00	0.03	0.02	0.02	0.01	0.06
patient_6	0.08	0.05	0.12	0.02	0.07	0.01	0.04	0.02	0.01	0.02	1.02
patient_7	0.01	0.07	0.17	0.03	0.06	0.00	0.09	0.04	0.03	0.04	1.41
patient_8	0.24	0.08	0.30	0.02	0.14	0.00	0.03	0.02	0.01	0.01	2.49
patient_9	0.11	0.01	0.31	0.03	0.13	0.00	0.06	0.04	0.03	0.00	0.95
patient_10	1.62	0.77	8.43	0.33	3.22	0.03	1.83	0.44	0.32	0.04	15.93
patient_11	0.09	0.02	0.58	0.04	0.18	0.00	0.17	0.04	0.04	0.00	1.29
patient_12	0.18	0.03	1.32	0.04	0.40	0.00	0.04	0.04	0.04	0.00	2.89
patient_13	3.43	3.20	16.33	0.29	3.65	0.58	2.58	1.34	1.75	0.76	26.83
patient_14	1.12	0.81	6.97	0.04	1.26	0.64	2.44	1.21	1.08	0.06	7.63
patient_15	3.28	4.03	13.55	0.20	4.49	0.42	319.27	1.55	2.36	0.12	15.88
patient_18	3.96	4.13	20.87	0.19	6.76	0.32	42.21	2.44	2.14	0.36	53.98
patient_19	0.07	0.06	0.35	0.04	0.10	0.00	0.37	0.03	0.05	0.04	0.27
patient_20	0.03	0.05	0.10	0.04	0.04	0.01	0.07	0.03	0.03	0.00	0.38
patient_21	1.39	2.21	9.49	0.23	5.69	0.23	11.88	2.58	1.56	0.51	11.99
patient_23	0.15	0.00	0.15	0.02	0.10	0.01	0.07	0.02	0.03	0.00	0.39
patient_24	3.12	1.00	5.84	0.33	7.56	0.66	4.23	3.60	1.52	0.88	3.32
patient_25	0.03	0.07	0.26	0.03	0.15	0.02	0.06	0.03	0.05	0.00	2.47
patient_26	15.88	1.60	27.11	0.32	10.18	0.77	2.40	4.03	0.34	0.49	161.07
patient_27	1.11	0.04	6.62	0.28	5.64	0.41	8.84	2.91	1.72	0.88	13.85
patient_28	0.02	0.05	0.27	0.03	0.12	0.00	0.17	0.04	0.03	0.09	0.25
patient_29	0.14	0.05	0.69	0.02	0.08	0.01	0.06	0.04	0.00	0.13	0.98
patient_30	0.24	0.06	0.18	0.01	0.10	0.01	0.06	0.04	0.01	0.05	0.87
patient_31	0.88	0.10	0.93	0.02	0.23	0.04	0.08	0.09	0.01	0.04	8.17
patient_32	5.05	0.88	9.91	0.16	2.96	0.40	14.68	1.12	1.36	0.11	41.95
patient_33	3.43	1.03	9.18	0.18	2.77	0.45	22.41	1.73	1.36	0.09	43.79
patient_34	0.04	0.05	0.13	0.01	0.05	0.00	0.05	0.05	0.01	0.00	0.04
patient_36	0.07	0.04	0.11	0.04	0.06	0.01	0.05	0.05	0.01	0.55	0.31
patient_37	1.20	0.67	8.83	0.09	2.86	0.01	15.04	3.32	1.21	0.82	5.29
patient_38	0.76	0.14	2.02	0.04	0.33	0.04	0.06	0.05	0.02	0.02	4.85
patient_39	0.63	0.11	1.06	0.04	0.23	0.03	0.10	0.06	0.02	0.98	0.36
patient_40	0.22	0.04	0.36	0.04	0.07	0.01	0.06	0.08	0.02	0.02	0.39

	mir375/mir16	mir375/mir20a	mir375/mir21	mir375/mir26b	mir375/mir93	mir375/mir106b	mir375/mir125b	mir375/mir141	mir375/mir200b	mir375/mir205	mir375/mir451a
patient_1	0.48	0.90	14.45	0.28	1.31	3.12	86.32	0.80	0.02	13.82	2.33
patient_2	0.55	2.24	13.45	1.05	1.61	2.47	160.41	1.42	0.00	19.99	9.64
patient_3	0.00	0.01	3.43	0.09	0.36	25.81	39.49	0.03	3.94	0.03	22.34
patient_4	5.16	1.63	29.12	0.81	5.80	4.81	98.69	0.98	0.02	5.00	56.03
patient_5	0.48	7.47	11.29	2.92	6.51	0.06	6.31	3.71	3.10	188.33	10.62
patient_6	3.11	2.02	5.05	0.67	3.07	0.34	1.63	0.98	0.59	41.16	42.09
patient_7	0.16	1.58	3.78	0.69	1.31	0.01	2.10	0.81	0.76	22.44	31.75
patient_8	20.24	6.92	24.77	1.75	11.38	0.09	2.40	1.32	0.67	83.53	207.61
patient_9	29.00	3.10	83.47	8.19	33.54	1.23	16.61	11.40	8.37	267.51	254.39
patient_10	40.35	19.20	209.74	8.27	79.97	0.64	45.55	10.91	8.03	24.87	396.31
patient_11	409.94	92.75	2739.32	185.17	846.06	7.64	820.07	196.65	182.91	4758.96	6130.15
patient_12	42.56	6.72	317.53	9.33	95.54	0.73	9.39	9.21	8.71	239.77	693.81
patient_13	4.50	4.20	21.41	0.38	4.79	0.76	3.38	1.75	2.29	1.31	35.18
patient_14	19.69	14.20	122.23	0.64	22.01	11.27	42.70	21.20	18.99	17.53	133.68
patient_15	26.77	32.88	110.65	1.65	36.68	3.46	2606.94	12.63	19.23	8.17	129.69
patient_18	11.11	11.59	58.59	0.54	18.98	0.90	118.47	6.84	6.02	2.81	151.53
patient_19	1.84	1.41	8.71	0.87	2.53	0.05	9.16	0.73	1.23	24.93	6.85
patient_20	6.45	12.80	22.94	8.25	8.58	1.88	15.81	6.76	6.45	233.43	88.66
patient_21	2.72	4.32	18.53	0.45	11.12	0.44	23.21	5.05	3.06	1.95	23.43
patient_23	68.96	2.17	67.34	8.05	42.86	4.26	33.14	9.53	14.64	449.76	173.94
patient_24	3.56	1.15	6.67	0.37	8.63	0.76	4.82	4.11	1.73	1.14	3.79
patient_25	14.57	31.00	112.19	13.52	65.83	10.49	24.73	13.04	20.35	430.39	1062.74
patient_26	32.67	3.30	55.76	0.65	20.94	1.59	4.93	8.28	0.70	2.06	331.26
patient_27	1.26	0.04	7.55	0.33	6.44	0.46	10.08	3.32	1.96	1.14	15.80
patient_28	0.22	0.52	2.94	0.27	1.26	0.02	1.84	0.40	0.28	10.79	2.69
patient_29	1.08	0.40	5.29	0.15	0.64	0.11	0.49	0.28	0.04	7.63	7.50
patient_30	4.97	1.17	3.78	0.26	1.98	0.23	1.24	0.88	0.13	20.64	17.89
patient_31	22.53	2.66	23.80	0.47	5.93	0.94	2.05	2.23	0.32	25.73	210.28
patient_32	46.98	8.23	92.26	1.46	27.54	3.71	136.64	10.44	12.70	9.31	390.48
patient_33	38.51	11.57	102.99	2.03	31.03	5.01	251.25	19.38	15.29	11.21	491.07
patient_34	18.60	24.13	66.95	7.65	26.20	1.71	24.09	25.69	4.63	528.23	20.51
patient_36	0.12	0.07	0.21	0.07	0.11	0.02	0.09	0.09	0.03	1.81	0.55
patient_37	1.46	0.81	10.74	0.11	3.48	0.02	18.30	4.04	1.48	1.22	6.44
patient_38	31.49	5.62	83.27	1.82	13.62	1.57	2.49	2.26	0.89	41.30	200.31
patient_39	0.64	0.12	1.08	0.04	0.23	0.03	0.10	0.06	0.02	1.02	0.37
patient_40	11.36	2.22	18.83	1.94	3.76	0.67	2.96	4.27	0.99	52.03	20.34

	mir451a/mir16	mir451a/mir20a	mir451a/mir21	mir451a/mir26b	mir451a/mir93	mir451a/mir106b	mir451a/mir125b	mir451a/mir141	mir451a/mir200b	mir451a/mir205	mir451a/mir375
patient_1	0.20	0.39	6.21	0.12	0.56	1.34	37.12	0.34	0.01	5.94	0.43
patient_2	0.06	0.23	1.40	0.11	0.17	0.26	16.65	0.15	0.00	2.07	0.10
patient_3	0.00	0.00	0.15	0.00	0.02	1.16	1.77	0.00	0.18	0.00	0.04
patient_4	0.09	0.03	0.52	0.01	0.10	0.09	1.76	0.02	0.00	0.09	0.02
patient_5	0.05	0.70	1.06	0.28	0.61	0.01	0.59	0.35	0.29	17.74	0.09
patient_6	0.07	0.05	0.12	0.02	0.07	0.01	0.04	0.02	0.01	0.98	0.02
patient_7	0.01	0.05	0.12	0.02	0.04	0.00	0.07	0.03	0.02	0.71	0.03
patient_8	0.10	0.03	0.12	0.01	0.05	0.00	0.01	0.01	0.00	0.40	0.00
patient_9	0.11	0.01	0.33	0.03	0.13	0.00	0.07	0.04	0.03	1.05	0.00
patient_10	0.10	0.05	0.53	0.02	0.20	0.00	0.11	0.03	0.02	0.06	0.00
patient_11	0.07	0.02	0.45	0.03	0.14	0.00	0.13	0.03	0.03	0.78	0.00
patient_12	0.06	0.01	0.46	0.01	0.14	0.00	0.01	0.01	0.01	0.35	0.00
patient_13	0.13	0.12	0.61	0.01	0.14	0.02	0.10	0.05	0.07	0.04	0.03
patient_14	0.15	0.11	0.91	0.00	0.16	0.08	0.32	0.16	0.14	0.13	0.01
patient_15	0.21	0.25	0.85	0.01	0.28	0.03	20.10	0.10	0.15	0.06	0.01
patient_18	0.07	0.08	0.39	0.00	0.13	0.01	0.78	0.05	0.04	0.02	0.01
patient_19	0.27	0.21	1.27	0.13	0.37	0.01	1.34	0.11	0.18	3.64	0.15
patient_20	0.07	0.14	0.26	0.09	0.10	0.02	0.18	0.08	0.07	2.63	0.01
patient_21	0.12	0.18	0.79	0.02	0.47	0.02	0.99	0.22	0.13	0.08	0.04
patient_23	0.40	0.01	0.39	0.05	0.25	0.02	0.19	0.05	0.08	2.59	0.01
patient_24	0.94	0.30	1.76	0.10	2.28	0.20	1.27	1.08	0.46	0.30	0.26
patient_25	0.01	0.03	0.11	0.01	0.06	0.01	0.02	0.01	0.02	0.40	0.00
patient_26	0.10	0.01	0.17	0.00	0.06	0.00	0.01	0.02	0.00	0.01	0.00
patient_27	0.08	0.00	0.48	0.02	0.41	0.03	0.64	0.21	0.12	0.07	0.06
patient_28	0.08	0.20	1.09	0.10	0.47	0.01	0.69	0.15	0.10	4.02	0.37
patient_29	0.14	0.05	0.70	0.02	0.09	0.01	0.06	0.04	0.00	1.02	0.13
patient_30	0.28	0.07	0.21	0.01	0.11	0.01	0.07	0.05	0.01	1.15	0.06
patient_31	0.11	0.01	0.11	0.00	0.03	0.00	0.01	0.01	0.00	0.12	0.00
patient_32	0.12	0.02	0.24	0.00	0.07	0.01	0.35	0.03	0.03	0.02	0.00
patient_33	0.08	0.02	0.21	0.00	0.06	0.01	0.51	0.04	0.03	0.02	0.00
patient_34	0.91	1.18	3.26	0.37	1.28	0.08	1.17	1.25	0.23	25.75	0.05
patient_36	0.22	0.12	0.37	0.13	0.20	0.03	0.16	0.17	0.05	3.26	1.80
patient_37	0.23	0.13	1.67	0.02	0.54	0.00	2.84	0.63	0.23	0.19	0.16
patient_38	0.16	0.03	0.42	0.01	0.07	0.01	0.01	0.01	0.00	0.21	0.00
patient_39	1.73	0.31	2.91	0.11	0.63	0.07	0.27	0.16	0.07	2.74	2.70
patient_40	0.56	0.11	0.93	0.10	0.18	0.03	0.15	0.21	0.05	2.56	0.05

	mir16/mir20a	mir16/mir21	mir16/mir26b	mir16/mir93	mir16/mir106b	mir16/mir125b	mir16/mir141	mir16/mir200b	mir16/mir205	mir16/mir375	mir16/mir451a
donor_1	0.27	3.97	0.05	0.75	0.02	0.00	0.00	0.19	0.70	1.03	15.72
donor_2	0.31	6.56	0.08	2.23	0.04	0.01	0.02	0.29	0.59	0.50	12.20
donor_3	0.37	4.19	0.13	1.17	0.05	0.03	0.00	0.28	0.75	1.47	21.42
donor_4	0.35	6.05	0.14	1.60	0.17	0.00	0.00	0.31	0.55	1.11	15.31
donor_5	0.20	1.44	0.04	0.46	0.04	0.46	0.17	0.04	0.09	0.67	9.81
donor_6	0.30	2.52	0.13	0.37	0.89	0.95	0.12	0.21	0.09	0.41	3.80
donor_7	0.48	2.80	0.10	0.61	0.10	1.66	0.28	0.15	0.17	0.36	9.53
donor_8	21.07	197.80	5.43	61.40	0.18	86.39	27.56	8.73	15.78	40.39	299.52
donor_9	0.09	4.18	0.04	0.27	0.00	0.90	0.49	0.11	0.26	0.52	4.98
donor_10	0.34	8.32	0.05	0.85	0.01	10.03	2.84	0.72	2.39	0.22	6.39
donor_11	0.22	3.51	0.04	0.94	0.01	3.19	2.21	0.32	0.91	2.13	8.30
donor_12	0.15	3.55	0.10	0.55	1485.73	2.97	7.81	0.09	0.46	0.20	7.65
donor_13	0.34	6.42	0.02	3.38	0.07	0.88	0.35	0.91	1.32	3.24	6.45
donor_14	10.50	269.20	1.19	157.66	38.96	40.05	19.23	65.79	50.31	23.76	114.87
donor_15	0.48	5.55	0.11	3.50	1.49	2.44	0.95	1.63	2.50	4.61	0.30
donor_16	0.51	9.48	0.11	4.83	1.63	1.47	0.99	1.81	2.03	7.03	11.69
donor_17	0.94	7.56	0.32	12.08	4.88	1.79	1.46	3.46	2.40	2.51	3.10
donor_18	0.34	9.95	0.28	7.48	8.67	2.82	1.18	3.33	3.12	5.52	6.58

	mir20a/mir16	mir20a/mir21	mir20a/mir26b	mir20a/mir93	mir20a/mir106b	mir20a/mir125b	mir20a/mir141	mir20a/mir200b	mir20a/mir205	mir20a/mir375	mir20a/mir451a
donor_1	3.73	14.80	0.17	2.80	0.07	0.01	0.00	0.72	2.60	3.86	58.58
donor_2	3.26	21.38	0.26	7.26	0.14	0.02	0.06	0.94	1.91	1.63	39.78
donor_3	2.68	11.23	0.36	3.15	0.13	0.07	0.00	0.75	2.01	3.94	57.40
donor_4	2.87	17.35	0.41	4.58	0.50	0.01	0.00	0.90	1.59	3.19	43.88
donor_5	5.00	7.22	0.20	2.30	0.19	2.28	0.83	0.20	0.43	3.36	49.05
donor_6	3.36	8.45	0.45	1.24	2.98	3.19	0.41	0.71	0.30	1.36	12.76
donor_7	2.07	5.79	0.21	1.26	0.20	3.44	0.58	0.31	0.35	0.74	19.74
donor_8	0.05	9.39	0.26	2.91	0.01	4.10	1.31	0.41	0.75	1.92	14.21
donor_9	11.65	48.74	0.41	3.09	0.03	10.49	5.69	1.28	3.00	6.09	58.00
donor_10	2.96	24.60	0.14	2.50	0.03	29.66	8.39	2.13	7.07	0.65	18.89
donor_11	4.51	15.81	0.20	4.26	0.03	14.37	9.97	1.43	4.11	9.62	37.41
donor_12	6.62	23.54	0.69	3.63	9840.03	19.65	51.70	0.61	3.05	1.36	50.70
donor_13	2.92	18.73	0.05	9.87	0.21	2.58	1.03	2.65	3.85	9.46	18.82
donor_14	0.10	25.63	0.11	15.01	3.71	3.81	1.83	6.26	4.79	2.26	10.94
donor_15	2.08	11.56	0.24	7.29	3.11	5.07	1.98	3.39	5.21	9.59	0.63
donor_16	1.95	18.44	0.22	9.39	3.16	2.86	1.93	3.52	3.94	13.69	22.76
donor_17	1.07	8.08	0.34	12.92	5.22	1.91	1.56	3.70	2.57	2.69	3.32
donor_18	2.92	29.11	0.82	21.86	25.35	8.26	3.45	9.75	9.13	16.13	19.25

	mir21/mir16	mir21/mir20a	mir21/mir26b	mir21/mir93	mir21/mir106b	mir21/mir125b	mir21/mir141	mir21/mir200b	mir21/mir205	mir21/mir375	mir21/mir451a
donor_1	0.25	0.07	0.01	0.19	0.01	0.00	0.00	0.05	0.18	0.26	3.96
donor_2	0.15	0.05	0.01	0.34	0.01	0.00	0.00	0.04	0.09	0.08	1.86
donor_3	0.24	0.09	0.03	0.28	0.01	0.01	0.00	0.07	0.18	0.35	5.11
donor_4	0.17	0.06	0.02	0.26	0.03	0.00	0.00	0.05	0.09	0.18	2.53
donor_5	0.69	0.14	0.03	0.32	0.03	0.32	0.11	0.03	0.06	0.47	6.80
donor_6	0.40	0.12	0.05	0.15	0.35	0.38	0.05	0.08	0.04	0.16	1.51
donor_7	0.36	0.17	0.04	0.22	0.04	0.59	0.10	0.05	0.06	0.13	3.41
donor_8	0.01	0.11	0.03	0.31	0.00	0.44	0.14	0.04	0.08	0.20	1.51
donor_9	0.24	0.02	0.01	0.06	0.00	0.22	0.12	0.03	0.06	0.12	1.19
donor_10	0.12	0.04	0.01	0.10	0.00	1.21	0.34	0.09	0.29	0.03	0.77
donor_11	0.29	0.06	0.01	0.27	0.00	0.91	0.63	0.09	0.26	0.61	2.37
donor_12	0.28	0.04	0.03	0.15	417.97	0.83	2.20	0.03	0.13	0.06	2.15
donor_13	0.16	0.05	0.00	0.53	0.01	0.14	0.06	0.14	0.21	0.50	1.00
donor_14	0.00	0.04	0.00	0.59	0.14	0.15	0.07	0.24	0.19	0.09	0.43
donor_15	0.18	0.09	0.02	0.63	0.27	0.44	0.17	0.29	0.45	0.83	0.05
donor_16	0.11	0.05	0.01	0.51	0.17	0.15	0.10	0.19	0.21	0.74	1.23
donor_17	0.13	0.12	0.04	1.60	0.65	0.24	0.19	0.46	0.32	0.33	0.41
donor_18	0.10	0.03	0.03	0.75	0.87	0.28	0.12	0.33	0.31	0.55	0.66

	mir26b/mir16	mir26b/mir20a	mir26b/mir21	mir26b/mir93	mir26b/mir106b	mir26b/mir125b	mir26b/mir141	mir26b/mir200b	mir26b/mir205	mir26b/mir375	mir26b/mir451a
donor_1	21.88	5.87	86.91	16.47	0.44	0.07	0.02	4.25	15.28	22.64	343.91
donor_2	12.51	3.84	82.04	27.86	0.53	0.09	0.24	3.61	7.32	6.24	152.67
donor_3	7.50	2.80	31.42	8.81	0.37	0.19	0.01	2.11	5.61	11.01	160.61
donor_4	7.02	2.45	42.46	11.22	1.22	0.03	0.00	2.19	3.89	7.81	107.39
donor_5	25.07	5.01	36.19	11.52	0.95	11.46	4.15	1.02	2.16	16.86	245.96
donor_6	7.54	2.24	18.98	2.79	6.68	7.17	0.91	1.59	0.67	3.06	28.65
donor_7	10.10	4.87	28.23	6.13	0.99	16.78	2.81	1.53	1.70	3.62	96.22
donor_8	0.18	3.88	36.45	11.31	0.03	15.92	5.08	1.61	2.91	7.44	55.20
donor_9	28.25	2.43	118.24	7.51	0.08	25.44	13.80	3.11	7.28	14.76	140.69
donor_10	20.65	6.98	171.76	17.46	0.22	207.06	58.55	14.86	49.37	4.54	131.87
donor_11	22.50	4.99	78.88	21.25	0.13	71.70	49.73	7.15	20.53	48.00	186.66
donor_12	9.58	1.45	34.04	5.25	14227.37	28.41	74.76	0.88	4.40	1.96	73.30
donor_13	53.56	18.35	343.74	181.16	3.94	47.30	18.99	48.60	70.71	173.56	345.30
donor_14	0.84	8.83	226.24	132.50	32.74	33.66	16.16	55.29	42.28	19.97	96.53
donor_15	8.79	4.22	48.81	30.80	13.12	21.42	8.38	14.30	22.01	40.51	2.68
donor_16	8.77	4.50	83.09	42.30	14.26	12.88	8.68	15.88	17.75	61.66	102.51
donor_17	3.12	2.92	23.60	37.71	15.23	5.58	4.55	10.79	7.51	7.85	9.69
donor_18	3.58	1.23	35.67	26.79	31.06	10.12	4.23	11.94	11.19	19.77	23.58

	mir93/mir16	mir93/mir20a	mir93/mir21	mir93/mir26b	mir93/mir106b	mir93/mir125b	mir93/mir141	mir93/mir200b	mir93/mir205	mir93/mir375	mir93/mir451a
donor_1	1.33	0.36	5.28	0.06	0.03	0.00	0.00	0.26	0.93	1.37	20.89
donor_2	0.45	0.14	2.94	0.04	0.02	0.00	0.01	0.13	0.26	0.22	5.48
donor_3	0.85	0.32	3.57	0.11	0.04	0.02	0.00	0.24	0.64	1.25	18.23
donor_4	0.63	0.22	3.79	0.09	0.11	0.00	0.00	0.20	0.35	0.70	9.57
donor_5	2.18	0.44	3.14	0.09	0.08	0.99	0.36	0.09	0.19	1.46	21.34
donor_6	2.70	0.81	6.81	0.36	2.40	2.57	0.33	0.57	0.24	1.10	10.28
donor_7	1.65	0.80	4.61	0.16	0.16	2.74	0.46	0.25	0.28	0.59	15.71
donor_8	0.02	0.34	3.22	0.09	0.00	1.41	0.45	0.14	0.26	0.66	4.88
donor_9	3.76	0.32	15.75	0.13	0.01	3.39	1.84	0.41	0.97	1.97	18.74
donor_10	1.18	0.40	9.84	0.06	0.01	11.86	3.35	0.85	2.83	0.26	7.56
donor_11	1.06	0.23	3.71	0.05	0.01	3.37	2.34	0.34	0.97	2.26	8.78
donor_12	1.83	0.28	6.49	0.19	2712.34	5.42	14.25	0.17	0.84	0.37	13.97
donor_13	0.30	0.10	1.90	0.01	0.02	0.26	0.10	0.27	0.39	0.96	1.91
donor_14	0.01	0.07	1.71	0.01	0.25	0.25	0.12	0.42	0.32	0.15	0.73
donor_15	0.29	0.14	1.58	0.03	0.43	0.70	0.27	0.46	0.71	1.32	0.09
donor_16	0.21	0.11	1.96	0.02	0.34	0.30	0.21	0.38	0.42	1.46	2.42
donor_17	0.08	0.08	0.63	0.03	0.40	0.15	0.12	0.29	0.20	0.21	0.26
donor_18	0.13	0.05	1.33	0.04	1.16	0.38	0.16	0.45	0.42	0.74	0.88

	mir106b/mir16	mir106b/mir20a	mir106b/mir21	mir106b/mir26b	mir106b/mir93	mir106b/mir125b	mir106b/mir141	mir106b/mir200b	mir106b/mir205	mir106b/mir375	mir106b/mir451a
donor_1	50.04	13.43	198.77	2.29	37.66	0.15	0.04	9.73	34.95	51.77	786.58
donor_2	23.40	7.18	153.43	1.87	52.11	0.17	0.44	6.75	13.70	11.67	285.53
donor_3	20.25	7.56	84.83	2.70	23.79	0.51	0.03	5.69	15.16	29.74	433.70
donor_4	5.74	2.00	34.75	0.82	9.18	0.02	0.00	1.79	3.18	6.39	87.88
donor_5	26.29	5.26	37.94	1.05	12.08	12.02	4.36	1.07	2.27	17.68	257.91
donor_6	1.13	0.34	2.84	0.15	0.42	1.07	0.14	0.24	0.10	0.46	4.29
donor_7	10.20	4.92	28.51	1.01	6.19	16.95	2.83	1.54	1.72	3.66	97.16
donor_8	5.71	120.23	1128.53	30.96	350.30	492.87	157.26	49.82	90.04	230.43	1708.94
donor_9	356.55	30.61	1492.05	12.62	94.74	321.03	174.16	39.28	91.87	186.28	1775.42
donor_10	94.25	31.87	784.12	4.57	79.69	945.29	267.29	67.85	225.38	20.74	602.04
donor_11	167.00	37.04	585.50	7.42	157.74	532.21	369.15	53.05	152.39	356.31	1385.60
donor_12	0.00	0.00	0.00	0.00	0.00	0.00	0.01	0.00	0.00	0.00	0.01
donor_13	13.60	4.66	87.25	0.25	45.98	12.01	4.82	12.34	17.95	44.05	87.65
donor_14	0.03	0.27	6.91	0.03	4.05	1.03	0.49	1.69	1.29	0.61	2.95
donor_15	0.67	0.32	3.72	0.08	2.35	1.63	0.64	1.09	1.68	3.09	0.20
donor_16	0.61	0.32	5.83	0.07	2.97	0.90	0.61	1.11	1.25	4.33	7.19
donor_17	0.20	0.19	1.55	0.07	2.48	0.37	0.30	0.71	0.49	0.52	0.64
donor_18	0.12	0.04	1.15	0.03	0.86	0.33	0.14	0.38	0.36	0.64	0.76

	mir125b/mir16	mir125b/mir20a	mir125b/mir21	mir125b/mir26b	mir125b/mir93	mir125b/mir106b	mir125b/mir141	mir125b/mir200b	mir125b/mir205	mir125b/mir375	mir125b/mir451a
donor_1	329.24	88.35	1307.94	15.05	247.81	6.58	0.25	64.00	230.00	340.66	5175.76
donor_2	140.21	43.00	919.29	11.21	312.20	5.99	2.63	40.42	82.08	69.91	1710.77
donor_3	39.34	14.68	164.78	5.25	46.20	1.94	0.06	11.05	29.44	57.77	842.40
donor_4	258.73	90.24	1565.72	36.87	413.57	45.06	0.16	80.85	143.52	287.82	3959.88
donor_5	2.19	0.44	3.16	0.09	1.01	0.08	0.36	0.09	0.19	1.47	21.46
donor_6	1.05	0.31	2.65	0.14	0.39	0.93	0.13	0.22	0.09	0.43	3.99
donor_7	0.60	0.29	1.68	0.06	0.36	0.06	0.17	0.09	0.10	0.22	5.73
donor_8	0.01	0.24	2.29	0.06	0.71	0.00	0.32	0.10	0.18	0.47	3.47
donor_9	1.11	0.10	4.65	0.04	0.30	0.00	0.54	0.12	0.29	0.58	5.53
donor_10	0.10	0.03	0.83	0.00	0.08	0.00	0.28	0.07	0.24	0.02	0.64
donor_11	0.31	0.07	1.10	0.01	0.30	0.00	0.69	0.10	0.29	0.67	2.60
donor_12	0.34	0.05	1.20	0.04	0.18	500.82	2.63	0.03	0.16	0.07	2.58
donor_13	1.13	0.39	7.27	0.02	3.83	0.08	0.40	1.03	1.49	3.67	7.30
donor_14	0.02	0.26	6.72	0.03	3.94	0.97	0.48	1.64	1.26	0.59	2.87
donor_15	0.41	0.20	2.28	0.05	1.44	0.61	0.39	0.67	1.03	1.89	0.13
donor_16	0.68	0.35	6.45	0.08	3.28	1.11	0.67	1.23	1.38	4.79	7.96
donor_17	0.56	0.52	4.23	0.18	6.76	2.73	0.82	1.93	1.35	1.41	1.74
donor_18	0.35	0.12	3.53	0.10	2.65	3.07	0.42	1.18	1.11	1.95	2.33

	mir141/mir16	mir141/mir20a	mir141/mir21	mir141/mir26b	mir141/mir93	mir141/mir106b	mir141/mir125b	mir141/mir200b	mir141/mir205	mir141/mir375	mir141/mir451a
donor_1	1331.47	357.30	5289.37	60.86	1002.15	26.61	4.04	258.84	930.12	1377.63	20931.02
donor_2	53.23	16.33	349.03	4.25	118.53	2.27	0.38	15.34	31.16	26.54	649.53
donor_3	651.81	243.18	2730.40	86.91	765.62	32.19	16.57	183.18	487.91	957.20	13958.78
donor_4	1586.58	553.36	9601.25	226.12	2536.11	276.33	6.13	495.76	880.09	1764.95	24282.70
donor_5	6.04	1.21	8.71	0.24	2.77	0.23	2.76	0.24	0.52	4.06	59.22
donor_6	8.26	2.46	20.81	1.10	3.06	7.33	7.86	1.74	0.74	3.35	31.42
donor_7	3.60	1.74	10.06	0.36	2.18	0.35	5.98	0.54	0.61	1.29	34.28
donor_8	0.04	0.76	7.18	0.20	2.23	0.01	3.13	0.32	0.57	1.47	10.87
donor_9	2.05	0.18	8.57	0.07	0.54	0.01	1.84	0.23	0.53	1.07	10.19
donor_10	0.35	0.12	2.93	0.02	0.30	0.00	3.54	0.25	0.84	0.08	2.25
donor_11	0.45	0.10	1.59	0.02	0.43	0.00	1.44	0.14	0.41	0.97	3.75
donor_12	0.13	0.02	0.46	0.01	0.07	190.31	0.38	0.01	0.06	0.03	0.98
donor_13	2.82	0.97	18.10	0.05	9.54	0.21	2.49	2.56	3.72	9.14	18.19
donor_14	0.05	0.55	14.00	0.06	8.20	2.03	2.08	3.42	2.62	1.24	5.97
donor_15	1.05	0.50	5.83	0.12	3.68	1.57	2.56	1.71	2.63	4.84	0.32
donor_16	1.01	0.52	9.57	0.12	4.87	1.64	1.48	1.83	2.04	7.10	11.81
donor_17	0.69	0.64	5.18	0.22	8.28	3.35	1.23	2.37	1.65	1.72	2.13
donor_18	0.85	0.29	8.43	0.24	6.33	7.34	2.39	2.82	2.65	4.67	5.58

	mir200b/mir16	mir200b/mir20a	mir200b/mir21	mir200b/mir26b	mir200b/mir93	mir200b/mir106b	mir200b/mir125b	mir200b/mir141	mir200b/mir205	mir200b/mir375	mir200b/mir451a
donor_1	5.14	1.38	20.44	0.24	3.87	0.10	0.02	0.00	3.59	5.32	80.87
donor_2	3.47	1.06	22.75	0.28	7.72	0.15	0.02	0.07	2.03	1.73	42.33
donor_3	3.56	1.33	14.91	0.47	4.18	0.18	0.09	0.01	2.66	5.23	76.20
donor_4	3.20	1.12	19.37	0.46	5.12	0.56	0.01	0.00	1.78	3.56	48.98
donor_5	24.66	4.93	35.60	0.98	11.34	0.94	11.27	4.09	2.13	16.59	241.97
donor_6	4.74	1.41	11.94	0.63	1.75	4.20	4.51	0.57	0.42	1.93	18.03
donor_7	6.61	3.19	18.49	0.65	4.01	0.65	10.99	1.84	1.11	2.37	63.01
donor_8	0.11	2.41	22.65	0.62	7.03	0.02	9.89	3.16	1.81	4.62	34.30
donor_9	9.08	0.78	37.99	0.32	2.41	0.03	8.17	4.43	2.34	4.74	45.20
donor_10	1.39	0.47	11.56	0.07	1.17	0.01	13.93	3.94	3.32	0.31	8.87
donor_11	3.15	0.70	11.04	0.14	2.97	0.02	10.03	6.96	2.87	6.72	26.12
donor_12	10.82	1.63	38.47	1.13	5.93	16078.91	32.11	84.49	4.98	2.22	82.84
donor_13	1.10	0.38	7.07	0.02	3.73	0.08	0.97	0.39	1.45	3.57	7.10
donor_14	0.02	0.16	4.09	0.02	2.40	0.59	0.61	0.29	0.76	0.36	1.75
donor_15	0.61	0.30	3.41	0.07	2.15	0.92	1.50	0.59	1.54	2.83	0.19
donor_16	0.55	0.28	5.23	0.06	2.66	0.90	0.81	0.55	1.12	3.88	6.46
donor_17	0.29	0.27	2.19	0.09	3.49	1.41	0.52	0.42	0.70	0.73	0.90
donor_18	0.30	0.10	2.99	0.08	2.24	2.60	0.85	0.35	0.94	1.66	1.97
	mir205/mir16	mir205/mir20a	mir205/mir21	mir205/mir26b	mir205/mir93	mir205/mir106b	mir205/mir125b	mir205/mir141	mir205/mir200b	mir205/mir375	mir205/mir451a
donor_1	1.43	0.38	5.69	0.07	1.08	0.03	0.00	0.00	0.28	1.48	22.50
donor_2	1.71	0.52	11.20	0.14	3.80	0.07	0.01	0.03	0.49	0.85	20.84
donor_3	1.34	0.50	5.60	0.18	1.57	0.07	0.03	0.00	0.38	1.96	28.61
donor_4	1.80	0.63	10.91	0.26	2.88	0.31	0.01	0.00	0.56	2.01	27.59
donor_5	11.60	2.32	16.74	0.46	5.33	0.44	5.30	1.92	0.47	7.80	113.77
donor_6	11.19	3.33	28.18	1.48	4.14	9.92	10.65	1.35	2.36	4.54	42.53
donor_7	5.94	2.87	16.61	0.59	3.60	0.58	9.87	1.65	0.90	2.13	56.60
donor_8	0.06	1.34	12.53	0.34	3.89	0.01	5.47	1.75	0.55	2.56	18.98
donor_9	3.88	0.33	16.24	0.14	1.03	0.01	3.49	1.90	0.43	2.03	19.33
donor_10	0.42	0.14	3.48	0.02	0.35	0.00	4.19	1.19	0.30	0.09	2.67
donor_11	1.10	0.24	3.84	0.05	1.04	0.01	3.49	2.42	0.35	2.34	9.09
donor_12	2.17	0.33	7.73	0.23	1.19	3230.72	6.45	16.98	0.20	0.45	16.64
donor_13	0.76	0.26	4.86	0.01	2.56	0.06	0.67	0.27	0.69	2.45	4.88
donor_14	0.02	0.21	5.35	0.02	3.13	0.77	0.80	0.38	1.31	0.47	2.28
donor_15	0.40	0.19	2.22	0.05	1.40	0.60	0.97	0.38	0.65	1.84	0.12
donor_16	0.49	0.25	4.68	0.06	2.38	0.80	0.73	0.49	0.89	3.47	5.77
donor_17	0.42	0.39	3.14	0.13	5.02	2.03	0.74	0.61	1.44	1.04	1.29
donor_18	0.32	0.11	3.19	0.09	2.39	2.78	0.90	0.38	1.07	1.77	2.11

	mir375/mir16	mir375/mir20a	mir375/mir21	mir375/mir26b	mir375/mir93	mir375/mir106b	mir375/mir125b	mir375/mir141	mir375/mir200b	mir375/mir205	mir375/mir451a
donor_1	0.97	0.26	3.84	0.04	0.73	0.02	0.00	0.00	0.19	0.68	15.19
donor_2	2.01	0.62	13.15	0.16	4.47	0.09	0.01	0.04	0.58	1.17	24.47
donor_3	0.68	0.25	2.85	0.09	0.80	0.03	0.02	0.00	0.19	0.51	14.58
donor_4	0.90	0.31	5.44	0.13	1.44	0.16	0.00	0.00	0.28	0.50	13.76
donor_5	1.49	0.30	2.15	0.06	0.68	0.06	0.68	0.25	0.06	0.13	14.59
donor_6	2.46	0.73	6.20	0.33	0.91	2.18	2.34	0.30	0.52	0.22	9.36
donor_7	2.79	1.34	7.79	0.28	1.69	0.27	4.63	0.77	0.42	0.47	26.55
donor_8	0.02	0.52	4.90	0.13	1.52	0.00	2.14	0.68	0.22	0.39	7.42
donor_9	1.91	0.16	8.01	0.07	0.51	0.01	1.72	0.93	0.21	0.49	9.53
donor_10	4.54	1.54	37.81	0.22	3.84	0.05	45.58	12.89	3.27	10.87	29.03
donor_11	0.47	0.10	1.64	0.02	0.44	0.00	1.49	1.04	0.15	0.43	3.89
donor_12	4.88	0.74	17.36	0.51	2.67	7255.02	14.49	38.12	0.45	2.25	37.38
donor_13	0.31	0.11	1.98	0.01	1.04	0.02	0.27	0.11	0.28	0.41	1.99
donor_14	0.04	0.44	11.33	0.05	6.64	1.64	1.69	0.81	2.77	2.12	4.83
donor_15	0.22	0.10	1.20	0.02	0.76	0.32	0.53	0.21	0.35	0.54	0.07
donor_16	0.14	0.07	1.35	0.02	0.69	0.23	0.21	0.14	0.26	0.29	1.66
donor_17	0.40	0.37	3.01	0.13	4.81	1.94	0.71	0.58	1.38	0.96	1.24
donor_18	0.18	0.06	1.80	0.05	1.36	1.57	0.51	0.21	0.60	0.57	1.19
	mir451a/mir16	mir451a/mir20a	mir451a/mir21	mir451a/mir26b	mir451a/mir93	mir451a/mir106b	mir451a/mir125b	mir451a/mir141	mir451a/mir200b	mir451a/mir205	mir451a/mir375
donor_1	0.06	0.02	0.25	0.00	0.05	0.00	0.00	0.00	0.01	0.04	0.07
donor_2	0.08	0.03	0.54	0.01	0.18	0.00	0.00	0.00	0.02	0.05	0.04
donor_3	0.05	0.02	0.20	0.01	0.05	0.00	0.00	0.00	0.01	0.03	0.07
donor_4	0.07	0.02	0.40	0.01	0.10	0.01	0.00	0.00	0.02	0.04	0.07
donor_5	0.10	0.02	0.15	0.00	0.05	0.00	0.05	0.02	0.00	0.01	0.07
donor_6	0.26	0.08	0.66	0.03	0.10	0.23	0.25	0.03	0.06	0.02	0.11
donor_7	0.10	0.05	0.29	0.01	0.06	0.01	0.17	0.03	0.02	0.02	0.04
donor_8	0.00	0.07	0.66	0.02	0.20	0.00	0.29	0.09	0.03	0.05	0.13
donor_9	0.20	0.02	0.84	0.01	0.05	0.00	0.18	0.10	0.02	0.05	0.10
donor_10	0.16	0.05	1.30	0.01	0.13	0.00	1.57	0.44	0.11	0.37	0.03
donor_11	0.12	0.03	0.42	0.01	0.11	0.00	0.38	0.27	0.04	0.11	0.26
donor_12	0.13	0.02	0.46	0.01	0.07	194.10	0.39	1.02	0.01	0.06	0.03
donor_13	0.16	0.05	1.00	0.00	0.52	0.01	0.14	0.05	0.14	0.20	0.50
donor_14	0.01	0.09	2.34	0.01	1.37	0.34	0.35	0.17	0.57	0.44	0.21
donor_15	3.28	1.58	18.23	0.37	11.50	4.90	8.00	3.13	5.34	8.22	15.13
donor_16	0.09	0.04	0.81	0.01	0.41	0.14	0.13	0.08	0.15	0.17	0.60
donor_17	0.32	0.30	2.43	0.10	3.89	1.57	0.58	0.47	1.11	0.77	0.81
donor_18	0.15	0.05	1.51	0.04	1.14	1.32	0.43	0.18	0.51	0.47	0.84

Supplementary Table S6. AUC values for all miRNA pairs (all patients and donors)

	AUC		AUC		AUC
mir205/mir375	0.925	mir20a/mir93	0.696	mir20a/mir451a	0.569
mir26b/mir375	0.911	mir205/mir16	0.696	mir125b/mir16	0.566
mir20a/mir375	0.871	mir205/mir26b	0.692	mir93/mir200b	0.564
mir125b/mir375	0.832	mir106b/mir375	0.687	mir451a/mir200b	0.563
mir21/mir375	0.808	mir141/mir200b	0.678	mir20a/mir141	0.556
mir93/mir375	0.800	mir141/mir106b	0.670	mir20a/mir16	0.556
mir141/mir375	0.794	mir125b/mir21	0.668	mir16/mir106b	0.553
mir16/mir375	0.789	mir125b/mir200b	0.667	mir93/mir21	0.550
mir26b/mir200b	0.775	mir26b/mir106b	0.654	mir93/mir106b	0.528
mir451a/mir375	0.762	mir26b/mir451a	0.651	mir125b/mir141	0.528
mir26b/mir21	0.749	mir125b/mir93	0.637	mir16/mir93	0.526
mir205/mir21	0.743	mir141/mir93	0.630	mir26b/mir125b	0.526
mir26b/mir93	0.743	mir205/mir141	0.623	mir205/mir125b	0.520
mir205/mir20a	0.734	mir26b/mir20a	0.621	mir451a/mir93	0.516
mir200b/mir375	0.734	mir26b/mir16	0.618	mir125b/mir20a	0.513
mir205/mir106b	0.730	mir26b/mir141	0.613	mir451a/mir141	0.510
mir205/mir93	0.728	mir16/mir200b	0.602	mir16/mir451a	0.507
mir205/mir200b	0.725	mir16/mir21	0.601	mir141/mir16	0.504
mir20a/mir200b	0.721	mir141/mir21	0.601	mir451a/mir106b	0.503
mir20a/mir21	0.715	mir451a/mir21	0.599	mir200b/mir21	0.503
mir205/mir451a	0.705	mir20a/mir106b	0.573	mir200b/mir106b	0.501
mir125b/mir106b	0.700	mir125b/mir451a	0.572	mir21/mir106b	0.500

Supplementary Table S7. Prognostic potency of amp-Ratio values of miRNA pairs

	Gleason score AUC		
	Gl-6 vs HD	Gl-7 vs HD	Gl-8/9 vs HD
Mir-205/mir-20a	0.625	0.739	0.935
Mir-26b/mir-21	0.644	0.761	0.917
Mir-26b/mir-93	0.699	0.736	0.852
Mir-451a/mir-375	0.699	0.778	0.833
	PSA level AUC		
	PSA<12 vs HD	PSA>12 vs HD	
Mir-205/mi-r375	0.889	0.971	
Mir-20a/mir-375	0.817	0.938	
Mir-26b/mir-375	0.894	0.931	
Mir-21/mir-375	0.762	0.866	
	Prostate volume AUC		
	V<50 vs HD	V>50 vs HD	
Mir-26b/mir-375	0.891	0.936	
Mir-125b/mir-375	0.783	0.927	
Mir-21/mir-375	0.79	0.833	
Mir-141/mir-375	0.785	0.821	

List of references

1. Duan. M.; Long. Y.; Yang. C.; Wu. X.; Sun. Y.; Li. J.; Hu. X.; Lin. W.; Han. D.; Zhao. Y.; et al. Selection and characterization of DNA aptamer for metastatic prostate cancer recognition and tissue imaging. *Oncotarget* **2016**. *7*. doi:10.18632/oncotarget.9262.
2. Wang. Y.; Luo. Y.; Bing. T.; Chen. Z.; Lu. M.; Zhang. N.; Shangguan. D.; Gao. X. DNA aptamer evolved by cell-SELEX for recognition of prostate cancer. *PLoS One* **2014**. *9*. doi:10.1371/journal.pone.0100243.
3. Li. P.; Yu. X.; Han. W.; Kong. Y.; Bao. W.; Zhang. J.; Zhang. W.; Gu. Y. Ultrasensitive and Reversible Nanoplatform of Urinary Exosomes for Prostate Cancer Diagnosis. *ACS Sensors* **2019**. *4*. 1433–1441. doi:10.1021/acssensors.9b00621.
4. Li. B.; Liu. C.; Pan. W.; Shen. J.; Guo. J.; Luo. T.; Feng. J.; Situ. B.; An. T.; Zhang. Y.; et al. Facile fluorescent aptasensor using aggregation-induced emission luminogens for exosomal proteins profiling towards liquid biopsy. *Biosens. Bioelectron.* **2020**. *168*. doi:10.1016/j.bios.2020.112520.
5. Almasi. F.; Gargari. S.L.M.; Bitaraf. F.; Rasoulinejad. S. Development of a single stranded DNA aptamer as a molecular probe for LNCap cells using cell-SELEX. *Avicenna J. Med. Biotechnol.* **2016**. *8*.
6. Jin. D.; Yang. F.; Zhang. Y.; Liu. L.; Zhou. Y.; Wang. F.; Zhang. G.J. ExoAPP: Exosome-Oriented. Aptamer Nanoprobe-Enabled Surface Proteins Profiling and Detection. *Anal. Chem.* **2018**. *90*. doi:10.1021/acs.analchem.8b03959.
7. Yang. D.K.; Chen. L.C.; Lee. M.Y.; Hsu. C.H.; Chen. C.S. Selection of aptamers for fluorescent detection of alpha-methylacyl-CoA racemase by single-bead SELEX. *Biosens. Bioelectron.* **2014**. *62*. doi:10.1016/j.bios.2014.06.027.
8. Parekh. P.; Kamble. S.; Zhao. N.; Zeng. Z.; Portier. B.P.; Zu. Y. Immunotherapy of CD30-expressing lymphoma using a highly stable ssDNA aptamer. *Biomaterials* **2013**. *34*. doi:10.1016/j.biomaterials.2013.07.099.
9. Jiang. Z.; Woda. B.A.; Rock. K.L.; Xu. Y.; Savas. L.; Khan. A.; Pihan. G.; Cai. F.; Babcook. J.S.; Rathanaswami. P.; et al. P504S: a new molecular marker for the detection of prostate carcinoma. *Am. J. Surg. Pathol.* **2001**. *25*. 1397–1404. doi:10.1097/00000478-200111000-00007.
10. Cannistraci. A.; Di Pace. A.L.; De Maria. R.; Bonci. D. MicroRNA as New Tools for Prostate Cancer Risk Assessment and Therapeutic Intervention: Results from Clinical Data Set and Patients' Samples. *Biomed Res. Int.* 2014. *2014*.
11. Shin. S.; Park. Y.H.; Jung. S.H.; Jang. S.H.; Kim. M.Y.; Lee. J.Y.; Chung. Y. –J Urinary exosome microRNA signatures as a noninvasive prognostic biomarker for prostate cancer. *npj Genomic Med.* **2021**. *6*. doi:10.1038/s41525-021-00212-w.
12. Borkowetz. A.; Lohse-Fischer. A.; Scholze. J.; Lotzkat. U.; Thomas. C.; Wirth. M.P.; Fuessel. S.; Erdmann. K. Evaluation of micrornas as non-invasive diagnostic markers in urinary cells from patients with suspected prostate cancer. *Diagnostics* **2020**. *10*. doi:10.3390/diagnostics10080578.
13. Lin. H.M.; Castillo. L.; Mahon. K.L.; Chiam. K.; Lee. B.Y.; Nguyen. Q.; Boyer. M.J.; Stockler. M.R.; Pavlakis. N.; Marx. G.; et al. Circulating microRNAs are associated with docetaxel chemotherapy outcome in castration-resistant prostate cancer. *Br. J. Cancer* **2014**. *110*. doi:10.1038/bjc.2014.181.
14. Fredsøe. J.; Rasmussen. A.K.I.; Mouritzen. P.; Bjerre. M.T.; Østergren. P.; Fode. M.; Borre. M.; Sørensen. K.D. Profiling of circulating microRNAs in prostate cancer reveals diagnostic biomarker potential. *Diagnostics* **2020**. *10*. doi:10.3390/diagnostics10040188.
15. Stoen. M.J.; Andersen. S.; Rakaee. M.; Pedersen. M.I.; Ingebriktsen. L.M.; Donnem. T.; Lombardi. A.P.G.; Kilvaer. T.K.; Busund. L.T.R.; Richardsen. E. Overexpression of mir-20a-5p in tumor epithelium is an independent negative prognostic indicator in prostate cancer—a multi-institutional study. *Cancers (Basel).* **2021**. *13*. doi:10.3390/cancers13164096.
16. Vanacore. D.; Boccellino. M.; Rossetti. S.; Cavaliere. C.; D'Aniello. C.; Di Franco. R.; Romano. F.J.; Montanari. M.; La Mantia. E.; Piscitelli. R.; et al. Micrornas in prostate cancer: An overview. *Oncotarget* 2017. *8*.
17. Bolayırlı. I.M.; Önal. B.; Adıgüzel. M.; Konukoğlu. D.; Demirdağ. Ç.; Kurtuluş. E.M.; Türegün. F.A.; Uzun. H. THE CLINICAL SIGNIFICANCE OF CIRCULATING miR-21. miR-142. miR-143. AND miR-146A IN PATIENTS WITH PROSTATE CANCER. *J. Med. Biochem.* **2022**. *41*. doi:10.5937/jomb0-32046.
18. Porzycki. P.; Ciszkowicz. E.; Semik. M.; Tyrka. M. Combination of three miRNA (miR-141. miR-21. and miR-375) as potential diagnostic tool for prostate cancer recognition. *Int. Urol. Nephrol.* **2018**. *50*. doi:10.1007/s11255-018-1938-2.
19. Cochetti. G.; Poli. G.; Guelfi. G.; Boni. A.; Egidi. M.G.; Mearini. E. Different levels of serum

microRNAs in prostate cancer and benign prostatic hyperplasia: Evaluation of potential diagnostic and prognostic role. *Onco. Targets. Ther.* **2016**. *9*. doi:10.2147/OTT.S119027.
20. Zhang. S.; Liu. C.; Zou. X.; Geng. X.; Zhou. X.; Fan. X.C.; Zhu. D.; Zhang. H.; Zhu. W. Microrna panel in serum reveals novel diagnostic biomarkers for prostate cancer. *PeerJ* 2021. *9*.
21. Jin. W.; Fei. X.; Wang. X.; Chen. F.; Song. Y. Circulating miRNAs as Biomarkers for Prostate Cancer Diagnosis in Subjects with Benign Prostatic Hyperplasia. *J. Immunol. Res.* **2020**. *2020*. doi:10.1155/2020/5873056.
22. Yin. W.; Chen. J.; Wang. G.; Zhang. D. MicroRNA-106b functions as an oncogene and regulates tumor viability and metastasis by targeting LARP4B in prostate cancer. *Mol. Med. Rep.* **2019**. *20*. doi:10.3892/mmr.2019.10343.
23. Mitchell. P.S.; Parkin. R.K.; Kroh. E.M.; Fritz. B.R.; Wyman. S.K.; Pogosova-Agadjanyan. E.L.; Peterson. A.; Noteboom. J.; O'Briant. K.C.; Allen. A.; et al. Circulating microRNAs as stable blood-based markers for cancer detection. *Proc. Natl. Acad. Sci. U. S. A.* **2008**. *105*. doi:10.1073/pnas.0804549105.
24. Konoshenko. M.Y.; Lekchnov. E.A.; Bryzgunova. O.E.; Zaporozhchenko. I.A.; Yarmoschuk. S. V.; Pashkovskaya. O.A.; Pak. S. V.; Laktionov. P.P. The panel of 12 cell-free microRNAs as potential biomarkers in prostate neoplasms. *Diagnostics* **2020**. *10*. doi:10.3390/diagnostics10010038.
25. Labbé. M.; Hoey. C.; Ray. J.; Potiron. V.; Supiot. S.; Liu. S.K.; Fradin. D. MicroRNAs identified in prostate cancer: Correlative studies on response to ionizing radiation. *Mol. Cancer* 2020. *19*.
26. Fan. B.; Jin. X.; Ding. Q.; Cao. C.; Shi. Y.; Zhu. H.; Zhou. W. Expression of mir-451a in prostate cancer and its effect on prognosis. *Iran. J. Public Health* **2021**. *50*. doi:10.18502/ijph.v50i4.6002.
